# Tris(methylthio)methane produced by *Mortierella hyalina* affects sulfur homeostasis in *Arabidopsis*

**DOI:** 10.1038/s41598-022-16827-7

**Published:** 2022-08-20

**Authors:** Y.-H. Tseng, S. Bartram, M. Reichelt, S. S. Scholz, A. K. Meents, A. Ludwig, A. Mithöfer, R. Oelmüller

**Affiliations:** 1 Department of Plant Physiology, Matthias Schleiden Institute of Genetics, Bioinformatics and Molecular Botany, Friedrich-Schiller-University Jena, 07743 Jena, Germany; 2 Department of Natural Product Biosynthesis, Max Planck Institute for Chemical Ecology, 07745 Jena, Germany; 3 Department of Biochemistry, Max Planck Institute for Chemical Ecology, 07745 Jena, Germany; 4 Research Group Plant Defense Physiology, Max Planck Institute for Chemical Ecology, 07745 Jena, Germany

**Keywords:** Plant sciences, Plant physiology, Plant stress responses, Secondary metabolism

## Abstract

Microbial volatiles are important factors in symbiotic interactions with plants. *Mortierella hyalina* is a beneficial root-colonizing fungus with a garlic-like smell, and promotes growth of *Arabidopsis* seedlings. GC–MS analysis of the *M. hyalina* headspace and NMR analysis of the extracted essential oil identified the sulfur-containing volatile tris(methylthio)methane (TMTM) as the major compound. Incorporation of the sulfur from the fungal volatile into plant metabolism was shown by ^34^S labeling experiments. Under sulfur deficiency, TMTM down-regulated sulfur deficiency-responsive genes, prevented glucosinolate (GSL) and glutathione (GSH) diminishment, and sustained plant growth. However, excess TMTM led to accumulation of GSH and GSL and reduced plant growth. Since TMTM is not directly incorporated into cysteine, we propose that the volatile from *M. hyalina* influences the plant sulfur metabolism by interfering with the GSH metabolism, and alleviates sulfur imbalances under sulfur stress.

## Introduction

Sulfur is an indispensable macronutrient required for proper plant growth, development and physiology. It is first incorporated into cysteine, then further into methionine, glutathione (GSH), vitamins and cofactors, such as thiamine and biotin, to carry out important biochemical processes. Notable examples are the iron-sulfur (Fe-S) clusters which are required for electron transport in photosynthesis, reduction and assimilation of sulfur and nitrogen^1^. In *Brassicales*, assimilation of sulfur contributes to the biosynthesis of glucosinolates (GSL), which are essential defense molecules against herbivores and pathogens^2,3^. Although being classified as secondary metabolites, GSLs can hold up to 30% of total sulfur content in the plant body and serves as sulfur reservoir^4,5^.

In natural environments, microorganisms play an important role in providing sulfate (SO_4_^2−^), the primary sulfur source accessible, to roots for the biosynthesis of sulfur-containing compounds in plants. As early as in 1877, scientists already knew that elemental sulfur (S^0^) can be oxidized to sulfate, and microbes were thought to be an essential part of it^6^. It was few decades later that scientists isolated the S-oxidizing bacteria *Thiobacillus denitrificans* and *T. thioparus*, and showed that they produce sulfate from S^0^^6–8^. It is now known that microorganisms possess sulfatases to mineralize organic sulfur, thereby releasing sulfate into the rhizosphere^9,10^. Furthermore, fungi were shown to mobilize sulfate-esters and activate arylsulfatases under sulfur-limiting conditions^11–14^. Fungal symbionts are also crucial in supporting plants with sulfur, as mycorrhizal fungi promote sulfur uptake in maize, clover and tomato^15,16^. The expression of sulfate transporters in plants can also be influenced by mycorrhizal fungi, resulting in improved sulfur status in host plants under sulfur deficient condition^17^.

Volatile organic compounds (VOCs) from microorganisms present another possible route to provide sulfur to plants. Dimethyl disulfide (DMDS) is produced by the bacteria *Serratia odorifera* and *Bacillus spp.* B55. Under sulfur deficiency, DMDS can sustain plant growth and increase root branching^18^. Labeling experiment demonstrated that the S-containing volatile is taken up by the plants^18,19^. Compared to bacteria, much less is known about sulfur-containing volatiles produced by fungi^20^. Besides DMDS, mercaptoacetone, 3-methylsulfanylpropan-1-ol, benzothiazole, 2-acetylthiazole, 3,5-dimethyl-1,2,4-trithiolane, 5-(1-propynyl)-thiophen-2-carbaldehyde and sulfur dioxide (SO_2_) were identified from various fungal headspaces^20^. Not much is known about the mechanisms of their incorporation into the plant metabolism, but SO_2_ can cross cell membranes directly from the surrounding air and influence sulfur distribution within leaf tissue^21–23^.

Incorporation of sulfur is a multi-step process. It starts primarily with the assimilation of sulfate by sulfate transporters (SULTRs) in the root cells. SULTR1;1 and SULTR1;2 act as the primary sulfate transporters in roots. SULTR2;1 is located in the xylem and the pericycle and responsible for root-shoot sulfur transport^24–28^. Once the sulfate is in root tissue, it is incorporated alongside with ATP into adenosine-5′-phosphosulfate (APS) via the enzyme ATP sulfurylase (ATPS). APS serves as the branching point between primary and secondary metabolism. Through APS reductase, APS is transformed into sulfite (SO_3_^2−^), and subsequently reduced to sulfide (S^2−^) by sulfite reductase. With *O*-acetyl-serine(thiol)lyase (OASTL), sulfide is further incorporated into *O*-acetylserine (OAS) to form the amino acid cysteine for primary metabolism^29^. On the other hand, APS goes into secondary metabolism through APS kinase, which catalyzes the formation of 3'-phosphoadenosine-5'-phosphosulfate (PAPS). PAPS serves as the molecule required for the last step of glucosinolate biosynthesis^30^.

Sulfur assimilation and dynamics are highly regulated under sulfur deficiency. In *Arabidopsis*, *SULFUR LIMITATION1* (*SLIM1*) is a central regulator of sulfur deficiency^31^. The transcription factor of the EIL family induces the expression of genes for sulfur uptake transporters. Furthermore, upon sulfur deficiency, genes for GSL catabolism are stimulated while those for GSL biosynthesis are repressed, thereby releasing sulfur from the GSL storage for proper plant growth^31^. Correspondingly, mutants defect in *SLIM1* cannot respond to sulfur deficiency, and show reduced root growth^31^. Finally, *SULFUR DEFICIENT INDUCED* (*SDI*) *1* and *SDI2* are often used as marker genes to monitor sulfur deficiency^32^. SDI1 is localized in the nucleus, and can repress GSL biosynthesis by interacting with MYB28, a major transcription factor for aliphatic GSL biosynthesis^32–34^. All these components fine tune the sulfur status in the plant body to optimize plant competence in response to sulfur limitation.

*Mortierella hyalina* belongs to the phylum *Mucoromycota*. It possesses a distinctive garlic-like smell in synthetic culture. In the co-cultivation experiments with *Arabidopsis thaliana* seedlings, *M. hyalina* promoted plant growth^35^. Similar results were obtained from three other *Mortierella* strains with garlic-like smells, while the growth responses were less from two other strains which did not smell (Fig. S1). In this study, we address the question whether the volatile from *M. hyalina* interferes with the plant metabolism and might be involved in the regulation of sulfur stress. The headspace of *M. hyalina* was analyzed by GC–MS to identify VOCs which are potentially involved in plant nutrition. By NMR, a sulfur-containing volatile, tris(methylthio)methane (TMTM; CAS Number 5418-86-0), was identified as the major chemical in the fungal headspace. Incorporation of the sulfur from the fungal volatile into plant metabolism was shown with stable sulfur isotope labeling experiments. Under sulfur deficiency, TMTM restored plant growth, reduced the consumption of sulfur-containing metabolites, and reduced the response of seedlings to sulfur deficiency. We propose that TMTM maintains sulfur homeostasis in the plant under sulfur limitation condition. Finally, biochemical analyses examining cysteine biosynthesis did not show direct incorporation of TMTM into *O*-acetylserine (OAS), suggesting that additional biochemical steps are involved before the sulfur from TMTM is incorporated into cysteine, or non-canonical incorporation mechanisms different from sulfate assimilation are involved.

## Materials and methods

### Growth medium and conditions for seedlings and fungi

Seeds of wild-type *A. thaliana* (ecotype Columbia-0), and *slim1*^31^ mutant were surface-sterilized for 8 min in the sterilization solution containing lauryl sarcosine (1%) and Clorix cleaner (23%). Surface-sterilized seeds were washed with sterilized water 8 times and placed on Petri dishes with MS medium supplemented with 0.3% gelrite^36^. The MS medium contains 1.5 mM MgSO_4_. After cold treatment at 4 °C for 48–72 h, plates were incubated at 22 °C under long day conditions (16 h light/ 8 h dark; 80 μmol m^−2^ s^−1^). Wild-type *A. thaliana* (ecotype Columbia-0) was curated in the lab. The *slim1* mutant was kindly provided by Prof. Dr. Stanislav Kopriva.

Sulfur deficiency assays were performed with MGRL medium^37^. 1 L of MGRL medium contains 1.75 mM NaH_2_PO_4_, 1.75 mM Na_2_HPO_4_, 2 mM Ca(NO_3_)_2_, 1.5 mM MgSO_4_, 3 mM KNO_3_, 67 µM Na_2_EDTA, 30 µM H_3_BO_3_, 10.3 µM MnSO_4_, 8.6 µM FeSO_4_, 1 µM ZnSO_4_, 1.0 µM CuSO_4_, 130 nM CoCl_2_, 24 nM (NH_4_)_6_Mo_7_O_24_, 1% sucrose, 0.3% Gelrite, pH 5.6. For MGRL medium with reduced sulfate, MgSO_4_ was replaced by MgCl_2_. The total sulfate concentration in high sulfate (HS) and low sulfate (LS) MGRL medium is 1520.9 µM and 20.9 µM, respectively.

*Mortierella* strains (*M. hyalina*, FSU-509; *M. alpina*, SF002698; *M. turficola*, SF009851; *M. vinacea*, SF002701; *M. longicollis*, SF009830) were obtained from Jena Microbial Resource Center (Jena, Germany). They were grown on Potato-Dextrose-Agar (PDA), pH 6.5, and at 23 °C in the dark^38^ for fresh subcultures and desiccator assays.

For the sulfur labeling assays, *M. hyalina* was grown on KM medium modified from^39^: 1 L of the medium contains 7.06 mM NaNO_3_, 6.98 mM KCl, 11.17 mM KH_2_PO_4_, 177.9 μM H_3_BO_3_, 6.4 μM CuCl_2_, 76.5 μM ZnCl_2_, 7.28 μM CoCl_2_, 0.89 μM (NH_4_)_6_Mo_7_O_24_, 29.6 μM MnCl_2_, 20 μM Na_2_EDTA, 20 μM FeCl_2_, 2% glucose, 0.2% peptone/trypton, 0.1% yeast extract, 0.1% casein hydrolysate, 1% agar. Finally, 2.11 mM ammonium sulfate ((NH_4_)_2_^32^SO_4_ or (NH_4_)_2_^34^SO_4_) was added to make the unlabeled (^32^S)/labeled (^34^S) medium, respectively.

Tris(methylthio)methane and ^34^S ammonium sulfate were purchased from Sigma-Aldrich (Germany).

### Desiccator assay and sulfur labeling experiment

Twenty-one 10-days old *Arabidopsis* seedlings germinated on MS were transferred to a Petri dish with sucrose-free MS medium (14.5 cm in diameter). In a 2.5 L desiccator, a 7-days old fungal culture grown on PDA was placed at the bottom. A plastic inlay with openings was inserted in the middle of the desiccator, and the big Petri-dish with seedlings was placed on top of it. To ensure the sterility of the experiment, the seam of the desiccator was sealed with 3 M Micropore tape and two layers of Parafilm (Fig. S2). Fungus and seedlings were incubated at 22 °C under long day conditions (16 h light/8 h dark; 80 μmol m^−2^ s^−1^) for 14 days. Number of inflorescence (flower stalk) and shoot fresh weight were measured.

The same procedure was followed for the sulfur labeling experiments, in which a 7-days old *M. hyalina* culture grown on labeled/unlabeled KM medium and sixteen 10-days old *Arabidopsis* seedlings were used. Root and shoot tissues were collected after 14 days for analysis.

### Sulfur deficiency assay with MGRL agar medium

After germinating on MS medium for 5 days, seedlings were rinsed gently with sterilize water and transferred to MGRL agar medium with high (1520.9 µM) or low (20.9 µM) sulfate concentrations and grown for 7 days.

To measure the influence of TMTM on seedlings’ fresh and dry weights, a three-compartment Petri dish (92 mm in diameter, Sarstedt, Germany) was used. Two compartments were filled with MGRL agar medium, both containing either high or low sulfate concentrations. A sheet of sterilized paper was put in the third empty compartment, to which 10 µL sterilized water and a 10 µL mixture with 0, 10, 100 or 1000 µg TMTM dissolved in dichloromethane was applied.

For monitoring root growth, 5 days-old seedlings were transferred onto high or low sulfate MGRL agar medium on a square plate (100 × 100 × 20 mm; Sarstedt, Germany). A sheet of sterilized paper was put on to the bottom of the plate, and TMTM was applied onto it as described above. Plates were incubated vertically. Root length was measured directly after transfer and after 7 days of treatment.

### RNA isolation, primers and real-time quantitative PCR (qPCR)

RNA was extracted with TRIzo Reagent (Invitrogen, Germany) following the guideline provided by the manufacturer. Traces of DNA in the RNA samples were digested with TURBO Dnase (Thermo Fisher Scientific, Germany). cDNA synthesis was performed with Omniscript RT Kit (Qiagen, Germany), following manufacturer’s instructions.

Each 20 μL qPCR reaction contained 2 μL of 10× DreamTaq Buffer (Thermo Fisher Scientific, Germany), 0.2 mM dNTP, 0.5 μM forward and reverse primers, 40 ng cDNA, 1 μL 20 × Evagreen (Biotum, Germany) and 1.5 U of DreamTaq DNA Polymerase (Thermo Fisher Scientific, Germany). Real-time PCR reaction was conducted with CFX Connect Real-Time PCR Detection System (Bio-Rad, Germany). The initial denaturation step was set at 95 °C for 3 min, followed by 40 cycles of denaturation at 95 °C for 10 s, annealing at 60 °C for 50 s, and extension at 72 °C for 1 min. Melt curve analysis was performed by incubating at 95 °C for 10 s, 65 °C 5 s, and increase to 95 °C at 0.5 °C/5 s increment. Melt curve analysis showed a single peak for all genes analyzed. Values were normalized to the housekeeping gene *RPS18B* (AT1G34030) for gene expression analysis. Gene-specific primer pairs used in this study and the gene accession numbers are listed in Table S1.

### GC–MS analysis of *M. hyalina* headspace

Headspace volatiles of a slant culture of *M. hyalina* grown on potato dextrose agar (PDA) in a glass tube with stopper were collected 14 days after inoculation with a solid phase micro extraction (SPME) fiber (Aldrich, red fiber, 100 µm PDMS) over 2 h. As a control, the headspace of the medium alone (PDA) was collected.

SPME fibers were desorbed in the injection port of a GC at 220 °C in splitless mode and a helium flow of 1 mL/min through the chromatographic column connected. The volatiles were separated chromatographically on a ZB-5 ms column (30 m × 0.25 mm × 0.25 µm, Phenomenex) with an GC-oven temperature program starting at 45 °C for 2 min, then heating up to 220 °C with a rate of 10 °C/min, followed by a heating rate of 30 °C/min to 280 °C, and was maintained for 1.83 min. The column was connected to a time-of-flight mass spectrometer (GCT, Micromass) via a transfer line (280 °C). Ion source temperature was set to 250 °C and ionization energy was 70 eV. For high resolution MS (HR-MS), heptacosa was continuously streaming into the source and the calibrated HR-MS profile was locked at *m/z* 218.9856.

A mixture of *n*-alkanes C_8_–C_20_ in *n*-hexane (Aldrich) was measured before and after a sample sequence under the same conditions except for the injector split ratio (1:50). Retention times of the *n*-alkanes were used to calculate the retention index (RI) for each peak in the GC–MS chromatogram according to the method of^52^.

Compounds were identified based on their mass spectra (MS) in combination with their individual RIs in comparison to MS and RI database^40^ using MassFinder software^41^ in combination with NIST MS Search. Authentic reference compounds were used additionally for identification. For relative quantification, identified peaks of the GC–MS total ion chromatogram (TIC) were integrated.

### Identification of the S-containing volatile from *M. hyalina*

To identify the main S-containing volatile produced by *M. hyalina*, the compound needed to be enriched for further analysis. For that, *M. hyalina* was cultured in liquid PDA media for two weeks at 23 °C in the dark without shaking. The fungus mats produced on the surface of the media were collected (total FW ≈ 180 g), rinsed twice with tap water and cut into pieces. The fungus material was subjected to hydro-destillation to obtain the essential oil which was further analyzed by NMR and GC–MS.

### GSL analysis by HPLC–UV

Fresh seedlings (20 to 100 mg) were harvested, weighted and freeze-dried until constant weight and ground to fine powder. GSLs were extracted with 1 mL of 80% methanol solution containing 0.05 mM of Sinalbin as internal standard. After centrifugation, 700 µL of extract was loaded onto DEAE Sephadex A 25 columns and treated with arylsulfatase for desulfation (Sigma-Aldrich). The eluted desulfo-GSLs were separated using high performance liquid chromatography (Agilent 1100 HPLC system, Agilent Technologies) on a reversed phase column (Nucleodur Sphinx RP, 250 × 4.6 mm, 5 µm, Macherey–Nagel, Düren, Germany) with a water (A)–acetonitrile (B) gradient: 0–1.0 min, 1.5% B; 1.0–6.0 min, 1.5–5% B; 6.0–8.0 min, 5–7% B; 8.0–18.0 min, 7–21% B; 18.0–23.0 min, 21–29% B; 23.0–23.1 min, 29–100% B; 23.1–24.0 min 100% B and 24.1–28.0 min 1.5% B; flow 1.0 mL min^−1^. Detection was performed with a photodiode array detector and peaks were integrated at 229 nm. Desulfated GSLs were identified by comparison of their retention time and UV spectra to those of purified standards previously extracted from *A. thaliana*^42^. We used the following molar response factors for quantification of individual GSL relative to the internal standard Sinalbin: 2.0 for aliphatic GSLs and 0.5 for indolic GSLs^43^.

### Relative quantification of GSH by LC–MS/MS

Relative quantification of GSH was achieved on an Agilent 1200 series HPLC system (Agilent Technologies) coupled to a tandem mass spectrometer API 3200 (Applied Biosystems, Darmstadt, Germany) via electrospray ionization (ESI) in positive ionization mode. An aliquot of the raw extract from GSL analysis (see above) was injected. A Zorbax Eclipse XDB-C18 column (Agilent Technologies) was used for separation. 0.05% formic acid and acetonitrile were used as solvent A and B, respectively, at a flow rate of 1.1 mL/min with the following profile: 0–0.5 min, 3–15% B; 0.5–2.5 min, 15–85% B; 2.5–2.6 min, 85–100% B; 2.6–3.5 min, 100% B, 3.5–3.6 min, 100% B–3% B and 3.6–6.0 min 3% B. The MS parameters were optimized as follows: ion spray voltage, 5500 V; turbo gas temperature, 650 °C; collision gas, 3 psi; curtain gas, 35 psi; ion source gas 1, 60 psi; ion source gas 2, 60 psi. MRM for the parent ion–product ion was set as follows: *m/z* 308.1–179.1 (CE, 17 V; DP, 46 V) for GSH. Relative quantification was accomplished and expressed in relative peak area units of the LC–MS/MS signal per mg fresh weight.

### Determination of incorporation of ^34^S into plant GSH and GSLs by LC-ESI-Q-ToF–MS

To determinate the ^34^S incorporation into plant metabolites, ultra-high-performance liquid chromatography–electrospray ionization–high resolution mass spectrometry (UHPLC–ESI–HRMS) was performed with a Dionex Ultimate 3000 series UHPLC (Thermo Scientific, Germany) and a Bruker timsToF mass spectrometer (Bruker Daltonics, Bremen, Germany). UHPLC was done by applying a Zorbax Eclipse XDB-C18 column (100 mm × 2.1 mm, 1.8 µm, Agilent Technologies, Waldbronn, Germany) with a solvent system of 0.1% formic acid (A) and acetonitrile (B) at flow rate of 0.3 mL/min. The elution profile was as follows: 0 to 0.5 min, 5% B; 0.5 to 11.0 min, 5–60% B in A; 11.0 to 11.1 min, 60–100% B, 11.1 to 12.0 min, 100% B and 12.1 to 15.0 min 5% B. For the coupling of LC to MS, electrospray ionization (ESI) in positive and negative ionization mode, for GSH and GSL, respectively, was used. The mass spectrometer was set with the parameters: capillary voltage 4.5/3.5 kV, end plate offset of 500 V, nebulizer pressure 2.8 bar, nitrogen at 280 °C at flow rate of 8 L/min as drying gas. Acquisition was conducted at 12 Hz with a mass range from *m/z* 50 to 1500. At the beginning of each chromatographic analysis, 10 µL of a sodium formate-isopropanol solution (10 mM solution of NaOH in 50/50 (v/v%) isopropanol- water containing 0.2% formic acid) was injected into the dead volume for recalibration of the mass spectrometer with the expected cluster ion m/z values. Peak areas were integrated from extracted ion chromatogram traces of the monoisotopic molecular ion peak ([M + H]^+^, [M − H]^−^) and of the isotopologues that could be detected with an isolation width of *m/z* ± 0.002. Details of *m/z* values of isotopologues are listed in Table S2. First we calculated the percentage of single isotopologues (% isotopologue) as a proportion of the sum of all isotopologues for each single compound (i.e. % of the monoisotopic molecular ion peak = peak area of the monoisotopic molecular ion peak × 100%/(peak area of the monoisotopic molecular ion peak + (peak area of “isotopologue + 1”) + (peak area of “isotopologue + 2”) + (peak area of “isotopologue + 3”) + (peak area of “isotopologue + 4”). In order to determine the incorporation of ^34^S, the ^34^S/^32^S ratio was calculated (^34^S/^32^S ratio = % “isotopologue + 2”/% of the monoisotopic molecular ion peak).

### Determination of incorporation of ^34^S into plant amino acids by GC-Orbitrap-HR-MS analysis

l-Methionine and l-Cysteine standards were obtained from Sigma-Aldrich (Taufkirchen, Germany). Stock standard solutions of each individual amino acid were prepared in a methanol/NH_4_OH (8 M) (1:1, v:v) buffer and stored at −20 °C. Methyl chloroformate and pyridine were obtained from Sigma-Aldrich, chloroform and HCl from Carl Roth GmbH (Karlsruhe, Germany).

Total protein hydrolysis, amino acid extraction and derivatization followed a protocol by^44^ with some modifications. In short: 15 to 35 mg plant material (fresh weight) suspended in 1 mL HCl (10 mM) was homogenized with a TissueLyser II (Quiagen GmbH, Hilden, Germany) using tungsten carbide balls (2 × 3 min at 25 Hz). After vortexing for 30 min at 37 °C and centrifugation at 20,000*g*, the supernatant was added to a strong cation exchange solid phase extraction cartridge (HyperSep SCX, bed weight: 50 mg, volume 1 mL (60,108–420; Thermo Fisher Scientific, Darmstadt, Germany)), pre-activated with 1 mL 0.01 M HCl and 1 mL ultrapure water (3 x). After washing with methanol/water (80/20, v/v), the cartridge was eluted with 500 µL of freshly prepared methanol/NH_4_OH (8 M) (1:1, v:v) buffer.

A sample of 400 µL was evaporated to dryness in a gentle nitrogen stream and re-suspended in 50 µL of the elution buffer. 10 µL of pyridine were added and vortexed for 10 s followed by adding 10 µL of methyl chloroformate and mixed for 10 min. The derivatization products were extracted by adding 50 µL chloroform and 50 µL sodium bicarbonate solution (50 mM), followed by vortexing for 2 min. After phase separation the lower organic phase was transferred to a new glass vial insert and dried for 30 min with anhydrous sodium sulfate. The dried solution was transferred to a micro insert (100 µL) and stored at −80 °C for further GC–MS analysis.

Gas chromatography-Orbitrap-high resolution-mass spectrometry analyses were carried out on a Trace 1310 series GC with split/splitless injector coupled to a Q Exactive GC Orbitrap mass spectrometer (Thermo Fisher Scientific, Bremen, Germany). Separations were obtained with a fused silica capillary column (ZB SemiVolatiles, 30 mm × 25 mm, 0.25 µm + 10 m guard column, Zebron, Phenomenex, Aschaffenburg, Germany) using Helium at a flow rate of 1 mL∙min^−1^. The injection volume was 4 µL and the injector was operated in splitless with surge mode (25 kPa for 1 min followed by a split flow of 20 mL∙min^−1^) at 240 °C. Programmed GC oven temperature started at 70 °C for 3 min, raised at 25 °C∙min^-1^ to 280 °C, and held for 5 min with 16.4 min total GC run time. The transfer line temperature was set to 250 °C and the ion source was operated in positive EI mode at 300 °C and 70 eV ionization energy. Resolution of the Orbitrap was set to 120,000 at 200 Da and the acquisition started at 6.5 min with a mass range from 34 to 300 amu.

Data acquisition and evaluation was performed using Xcalibur (Thermo Fisher Scientific).

### OASTL activity assay monitoring cysteine biosynthesis

200 mg *Arabidopsis* wild-type *Col-0* leaves were homogenized in liquid nitrogen. 0.5 mL extraction buffer (50 mM HEPES–KOH, pH 7.5; 10 mM KCl; 1 mM EDTA; 1 mM EGTA; 30 mM DTT; 0.5 mM PMSF and 10% (v/v) glycerol) was added and mixed at 4 °C for 10 min with frequent shaking. After centrifugation at 16,000*g* for 10 min, supernatant was collected. Protein concentration was measured with ROTI Quant (Carl-Roth, Germany) following manufacturer’s instruction.

The OASTL activity assay was carried out in a volume of 0.1 mL containing 10 mg protein extract, 100 mM HEPES–KOH pH 7.5; 5 mM DTT; 10 mM OAS and 10 mM Na_2_S or 4 mM TMTM. The reaction was initiated by the addition of OAS, and was incubated for 10 min at 25 °C. Termination of the reaction was done by adding 50 μL of 20% (w/v) trichloroacetic acid followed by centrifugation at 12,500*g*.

100 µL of the supernatant was transferred to a new tube and incubated in 200 µL of 134 mM Tris–HCl, pH 8.0 and 1 mM DTT at room temperature for 30 min. The reduced sample was mixed with 200 µL acetic acid and 200 µL ninhydrin reagent (250 mg ninhydrin dissolved in 6 mL acetic acid and 4 mL concentrated HCl). The tube was incubated at 90 °C for 10 min, then cooled rapidly on ice for 2 min. The sample was diluted with 95% ethanol and measured at 560 nm to quantify the synthesized cysteine.

### Measurement of root length

Plates were scanned with an Epson scanner (Perfection V600 Photo, Epson, Germany). Files were imported into ImageJ^45^. Root length was measured by SmartRoot plug-in with semi-automated root tracing method^46^.

### Statistical tests

Statistical tests were performed using R studio version 1.1.463 with R version 3.4.4. Figures were plotted using Python 3.7.4 and arranged with LibreOffice Draw 5.1.6.2.

All the experiments were performed in accordance with relevant guidelines and regulations.

## Results

### *M. hyalina* produces the sulfur-containing volatile tris(methylthio)methane (TMTM)

**Figure 1:**
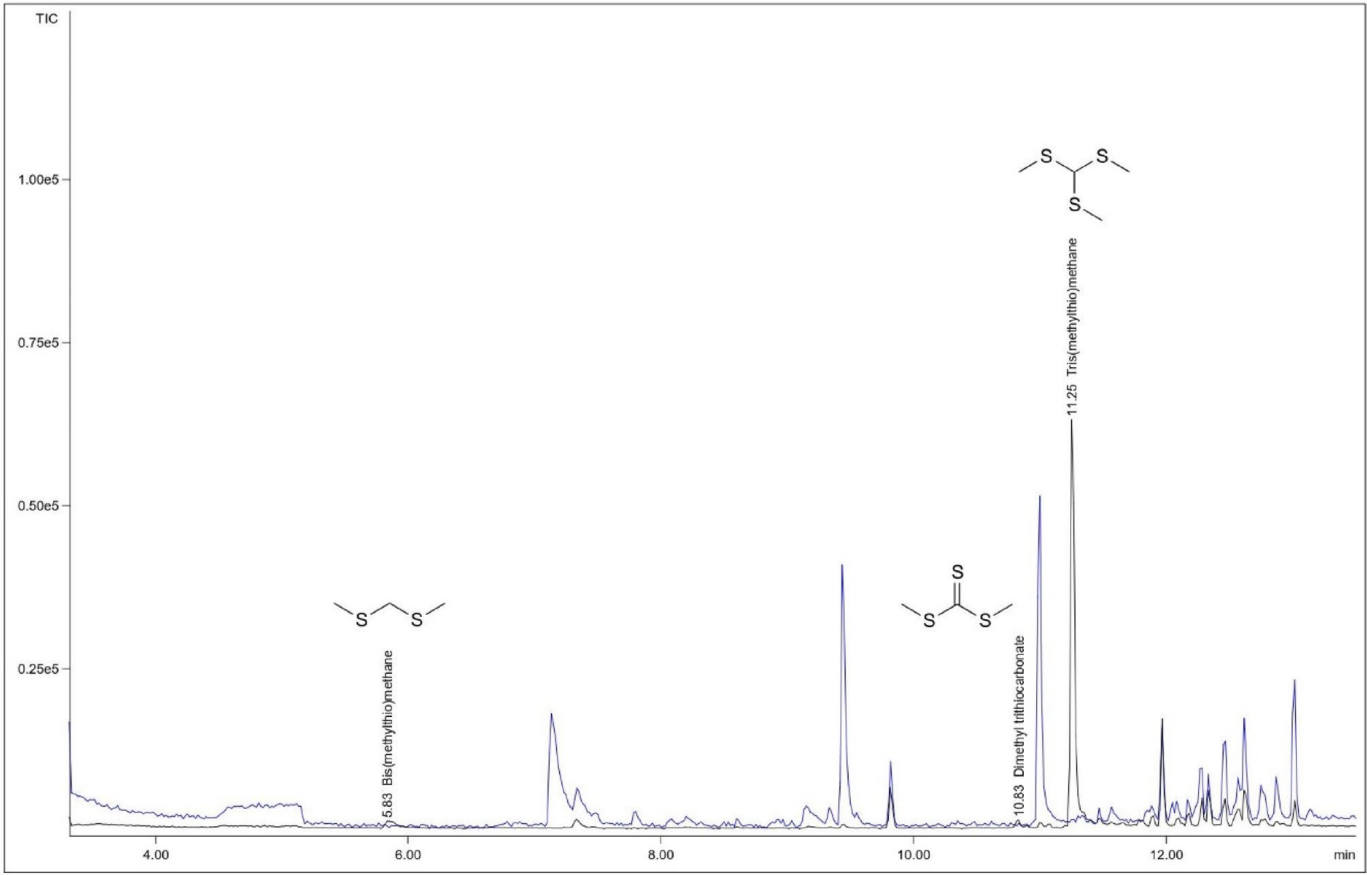
GC–MS chromatogram of the headspace of *M. hyalina* (black) and the growth medium alone (blue). Identified signals are not present in the headspace of the growth medium. The three strong signals in the chromatogram of the headspace of the growth medium could be identified by MS and RI as benzaldehyde (7.13 min) nonanal (9.44 min), and decanal (11.00 min).

To identify the volatiles from *M. hyalina* which are responsible for the garlic-like smell, the GC–MS chromatograms of SPME volatile collections of the headspace of slant cultures of *M. hyalina* were compared with the collections from the headspace of the growth medium. Three major constituents could be identified (Fig. 1) of which the HR-MS of the molecular ions M^+^ and [M + 2]^+^ (for the ^34^S isotopologue) revealed the molecular formulas C_3_H_8_S_2_ (*m/z* measured 108.0062, 110.0020 calc. 108.0062, 110.0020; RI 894; 2% rel.), C_3_H_8_S_3_ (*m/z* measured 137.9626, 139.9585 calc. 137.9626, 139.9584; RI 1197; 2% rel.), and C_4_H_10_S_3_ (*m/z* measured 153.9942, 155.9910 calc. 153.9939, 155.9897, RI 1217; 96% rel.). C_3_H_8_S_2_ and C_3_H_8_S_3_ could be identified as bis(methylthio)methane (RI_lit._ 889) and dimethyl trithiocarbonate (RI_lit._ 1196), respectively by comparison of their mass spectra and RI with the datasets of the NIST library and additionally with mass spectra and RI of authentic samples recorded under the same conditions.

For C_4_H_10_S_3_, the major compound of the headspace of *M. hyalina*, library searches in NIST and Wiley mass spectra databases revealed no hit in combination with the RI. Therefore *M. hyalina* was extracted by hydro distillation. The obtained essential oil consisted mainly of three compounds (by GC–MS): Octenol-3-ol (22.4%) 3-octenone (21.7%) and C_4_H_10_S_3_ (27.3%). NMR analysis of the mixture could reduce the structure motive of C_4_H_10_S_3_ to (CH_3_-X)_*n*_–CH (X = S, O, etc*.*) which in combination with the empirical formula C_4_H_10_S_3_ from HR-MS led to the structure of tris(thiomethyl)methane. Comparison with an authentic sample of tris(methylthio)methane (Aldrich) showed to be identical with respect to NMR and mass spectra, and RI (Fig. 1 and Table 1).

**Table 1:** Mass spectra and retention indices (RI) in comparison with authentic samples.

RT	Compound	Formula by HR-MS	CAS #	Retention index	RI (lit.)	Rel %	Authenic reference
5.83	Bis(methylthio)methane	C_3_H_8_S_2_	[1618-26-4]	894	889	2%	Y
10.83	Dimethyl trithiocarbonate	C_3_H_6_S_3_	[2314-48-9]	1197	1196	2%	Y
11.25	Tris(methylthio)methane	C_4_H_10_S_3_	[5418-86-0]	1217	^§^	98%	Y

^§^The RI given in the NIST MS database and by other authors as well as the mass spectrum of Tris(methylthio)methane published there and in the Wiley MS database are not correct. GC-MS of an authentic sample purchased from Aldrich revealed an RI of 1217 and a mass spectrum identical to the mass spectrum of the compound at the identical retention time from the headspace of *M. hyalina*.

### Sulfur atoms from TMTM are incorporated into plant metabolites

**Figure 2:**
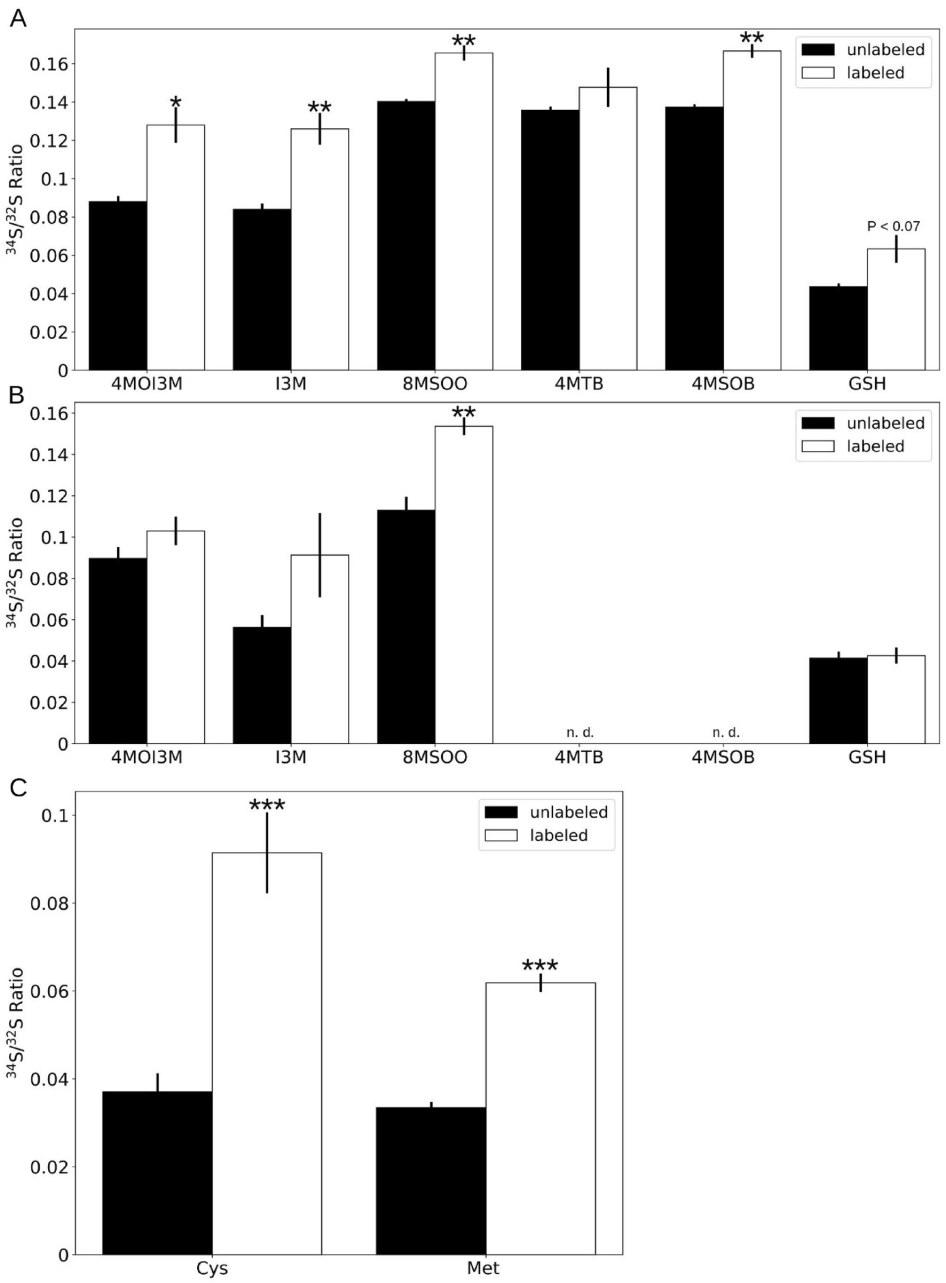
Sulfur atoms from fungal volatiles are incorporated into plant tissues. (**A,B**) ^34^S/^32^S ratios of glucosinolates and glutathione in shoot (**A**) and root (**B**) tissues. (**C**) ^34^S/^32^S ratio of cysteine (Cys) and methionine (Met) from *Arabidopsis* shoot. *M. hyalina* was grown on modified KM plates with the addition of ^32^S-ammonium sulfate (unlabeled) or ^34^S-ammonium sulfate (labeled), and co-cultivated with *Arabidopsis* seedlings in a desiccator. Error bars represent SEs from 3 biological replicates, each contains at least 3 technical replicates from 16 seedlings. Asterisks indicate significance level from Student’s t-test between unlabeled and labeled samples (*p < 0.05; **p < 0.01; ***p < 0.001). *n.d.* not detected.

To test whether sulfur from TMTM is incorporated into plant material, we grew *M. hyalina* on modified KM medium with the addition of ^32^S- or ^34^S-ammonium sulfate, and co-cultivated them together with *Arabidopsis* seedlings in the same desiccator without direct physical contact. After 14 days, shoot and root tissues were collected, and the ^34^S/^32^S ratio of GSLs and GSH was analyzed with LC–MS. For shoots, a significant increase in the ^34^S/^32^S ratios for the GSLs was detected (8.8% → 12.8% for 4MOI3M; 8.4% → 12.6% for I3M; 14% → 16.6% for 8MSOO; 13.7% → 16.7% for 4MSOB; Fig. 2). The ratio was also higher for GSH in the shoots (4.4% → 6.3%). With the exception of 8MSOO, for which we also observed a significant increase in the roots (11.3% → 15.4%), the increases for the other compounds were much less (4.1% → 4.3%; Fig. 2A,B; Figure S3 and S4).

As a proxy for the sulfur incorporation into *Arabidopsis* protein, we chose the sulfur containing amino acids cysteine and methionine from the total hydrolysate of *Arabidopsis* plant material. After derivatization, the relative amount of ^34^S was monitored by evaluating [M + 2]^+^ to [M]^+^ ratio of sulfur containing ions using GC-high resolution mass spectrometry.

Due to the fact that the molecular ions and their M + 2 isotopomers are not detectable (cysteine) or at a very low abundance (methionine), we chose the following sulfur containing fragments for the evaluation of relative ^34^S abundance: For cysteine [C_4_H_5_O_2_^32^S]^+^ *m/z*: 117.0005 vs. [C_4_H_5_O_2_^34^S]^+^ *m/z*: 118.9963; [C_4_H_6_O_2_N^32^S]^+^ *m/z*: 132.0114 vs. [C_4_H_6_O_2_N^34^S]^+^ *m/z*: 134.0072; [C_6_H_10_O_4_N^32^S]^+^ *m/z*: 192.0325 vs. [C_6_H_10_O_4_N^34^S]^+^ *m/z*: 194.0283 and for methionine [C_2_H_5_^32^S]^+^ *m/z*: 61.0106 vs. [C_2_H_5_^34^S]^+^ *m/z*: 63.0064; [C_6_H_12_O_2_N^32^S]^+^ *m/z*: 162.0583 vs. [C_6_H_12_O_2_N^34^S]^+^ *m/z*: 164.0541; [C_7_H_11_O_3_N^32^S]^+^ *m/z*: 189.0454 vs. [C_7_H_11_O_3_N^34^S]^+^ *m/z*: 191.0412, respectively (Figs. S5 and S6). The ^34^S/^32^S ratio given in percent of the respective ^34^S isotopomeric fragment was extracted from each GC–MS run by averaging over three MS scans at the respective retention time (9.76 min, cysteine derivative, 9.30 min, methionine derivative) in each run.

From three biological replicates, the mean ^34^S/^32^S ratios for the individual amino acids in plants under treatment with a substrate containing ^34^S at natural abundance *vs*. the ^34^S enriched substrate were 3.31% (SE ± 0.22) vs. 8.88% (SE ± 0.75) for cysteine (p < 0.001) and 3.84% (SE ± 0.38) vs. 6.19% (SE ± 0.33) for methionine (p < 0.001), respectively (Fig. 2C).

In conclusion, sulfur from *M. hyalina* headspace is incorporated into the plant amino acids, GSLs and GSH.

### TMTM influences plant growth under sulfur deficiency

**Figure 3:**
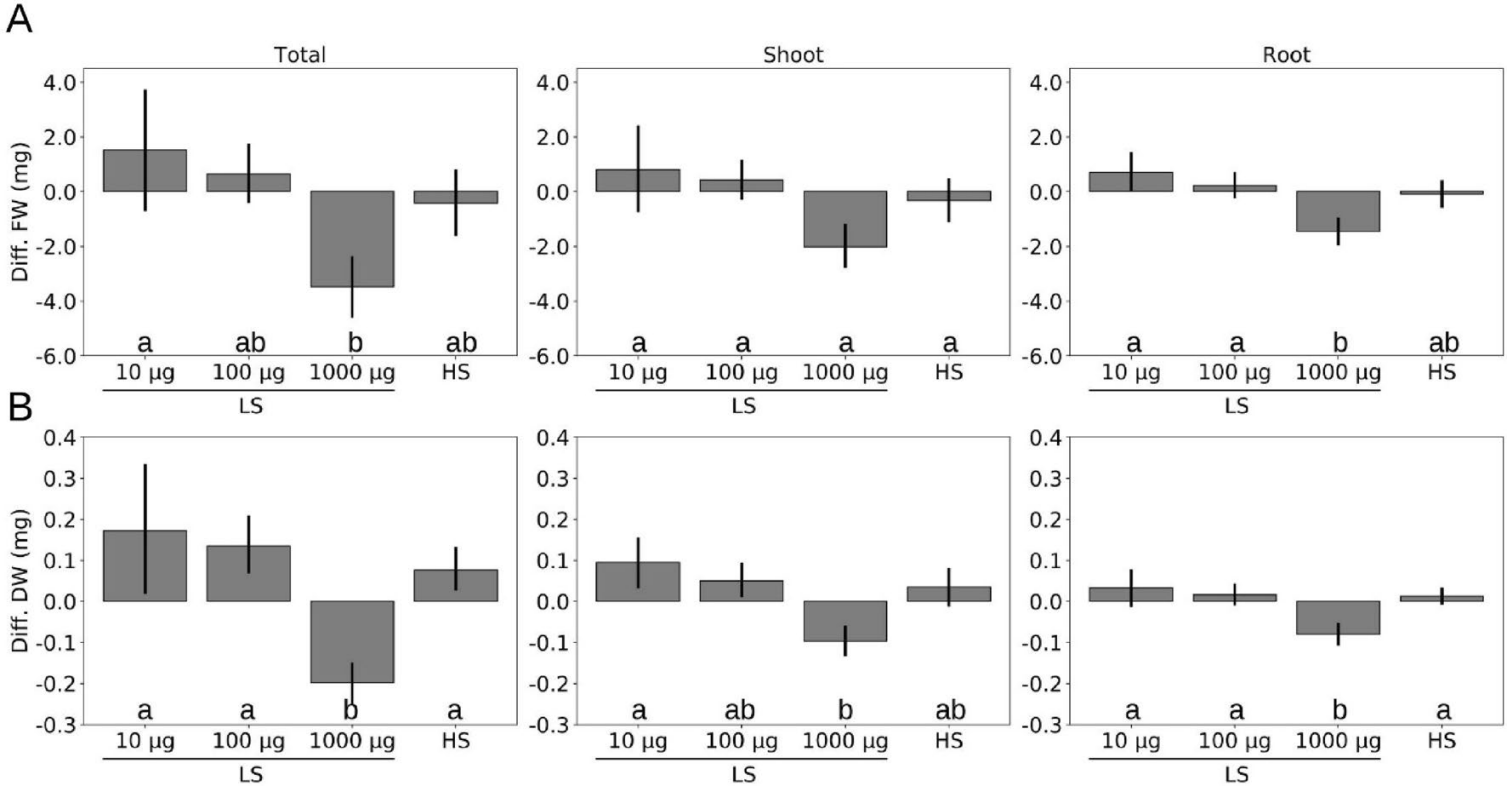
Fungal volatile influences *A. thaliana* growth under sulfur deficiency. (**A,B**) Differences in fresh weight (**A**) and in dry weight (**B**) of seedlings grown on high sulfate (HS) or on low sulfate (LS) MGRL medium with the addition of TMTM (10, 100 and 1000 µg) to seedlings grown on LS without TMTM. Error bars represent SEs from at least 5 independent biological replicates, each with 8 seedlings. Statistical significance was determined by Duncan’s multiple range test with p-value < 0.05, and indicated with lower-case alphabets.

Since TMTM contains sulfur, we tested the effect of the volatile on *Arabidopsis* plants. Five days-old seedlings were transferred to MGRL agar medium with either high sulfate (HS) or low sulfate (LS) concentrations and applied with 0, 10, 100 or 1000 µg TMTM. After 7 days, the fresh and dry weights of the total seedlings, the shoots and the roots were analyzed. Figure 3 shows the effects of TMTM on the weights of seedlings grown on LS in comparison to seedlings grown on HS. In all instances, TMTM had the strongest growth promoting effect for seedlings grown on LS supplemented with 10 or 100 µg TMTM (Fig. 3). The high dose of 1000 µg TMTM reduced plant growth. In summary, low doses of TMTM (10–100 µg) had positive effects on plant fresh and dry weights under sulfur deficiency, while the higher dose (1000 µg) had a negative effect.

We also tested whether TMTM promoted growth of seedlings grown on HS (cf. “Methods and materials”). Different doses of pure TMTM were applied to 10-days old seedlings grown on HS medium in desiccator for 1 or 2 weeks. Although the same trend was visible, the growth promoting effect of the volatile was not significant (data not shown).

### TMTM maintains root growth under sulfur deficiency

**Figure 4:**
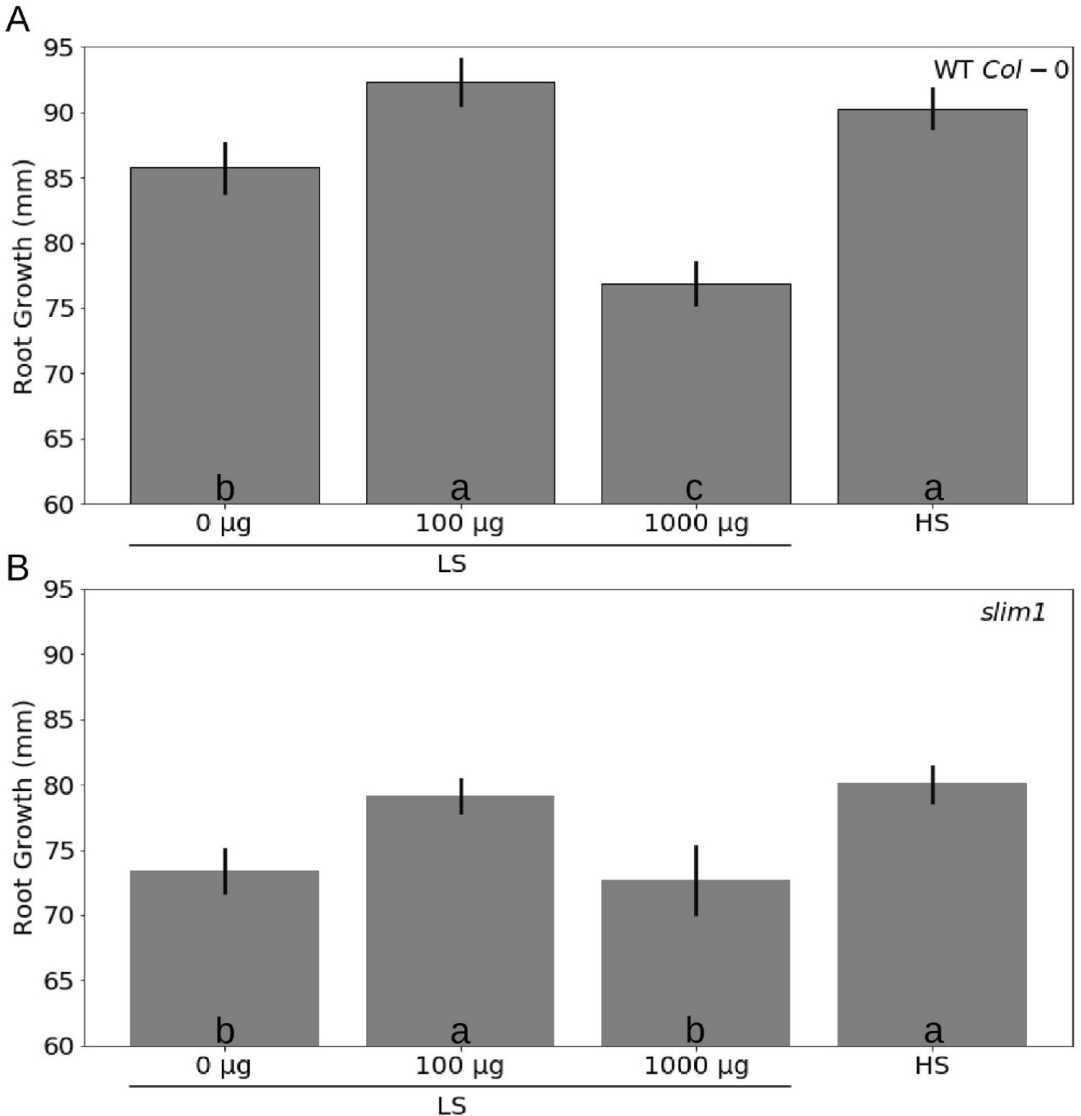
TMTM contributes positively to root growth. Wild-type (**A**) or *slim1* (**B**) seedlings’ root growth on high sulfate medium (HS) or on low sulfate medium (LS) with addition of 0, 100 or 1000 µg TMTM was measured 7 days after application. Error bars represent SEs from at least 6 biological replicates for wild-type and 8 biological replicates for *slim1*. Statistical significance was determined by Duncan’s multiple range test with p-value < 0.05, and indicated with lower-case letters.

To examine whether TMTM affects the root growth under sulfur deficiency, 5-days old seedlings of wild-type (*Col-0*) and *slim1*, a mutant which fails to respond to sulfur deficiency^31^, were transferred to LS medium and grown vertically for additional 7 days. Figure 4 shows the increase in the root lengths after 7 days. Compared to LS condition without TMTM, the root lengths of both wild-type (WT) and *slim1* seedlings were significantly higher when they were exposed to 100 µg TMTM (≈ 9% and 7% increase for WT and *slim1*, respectively; Fig. 4) and reached the level of seedlings which were grown on HS medium without the volatile. In accordance with the fresh and dry weight data, addition of 1000 µg TMTM reduced the root growth rate in WT for about 10% (Fig. 4A). Interestingly, the reduction in root growth was not affected in *slim1* (≈ 1% reduction compared to LS without TMTM; Fig. 4B). The differences might be due to an effect of TMTM on the sulfur homeostasis.

### TMTM reduces sulfur deficiency responses

**Figure 5:**
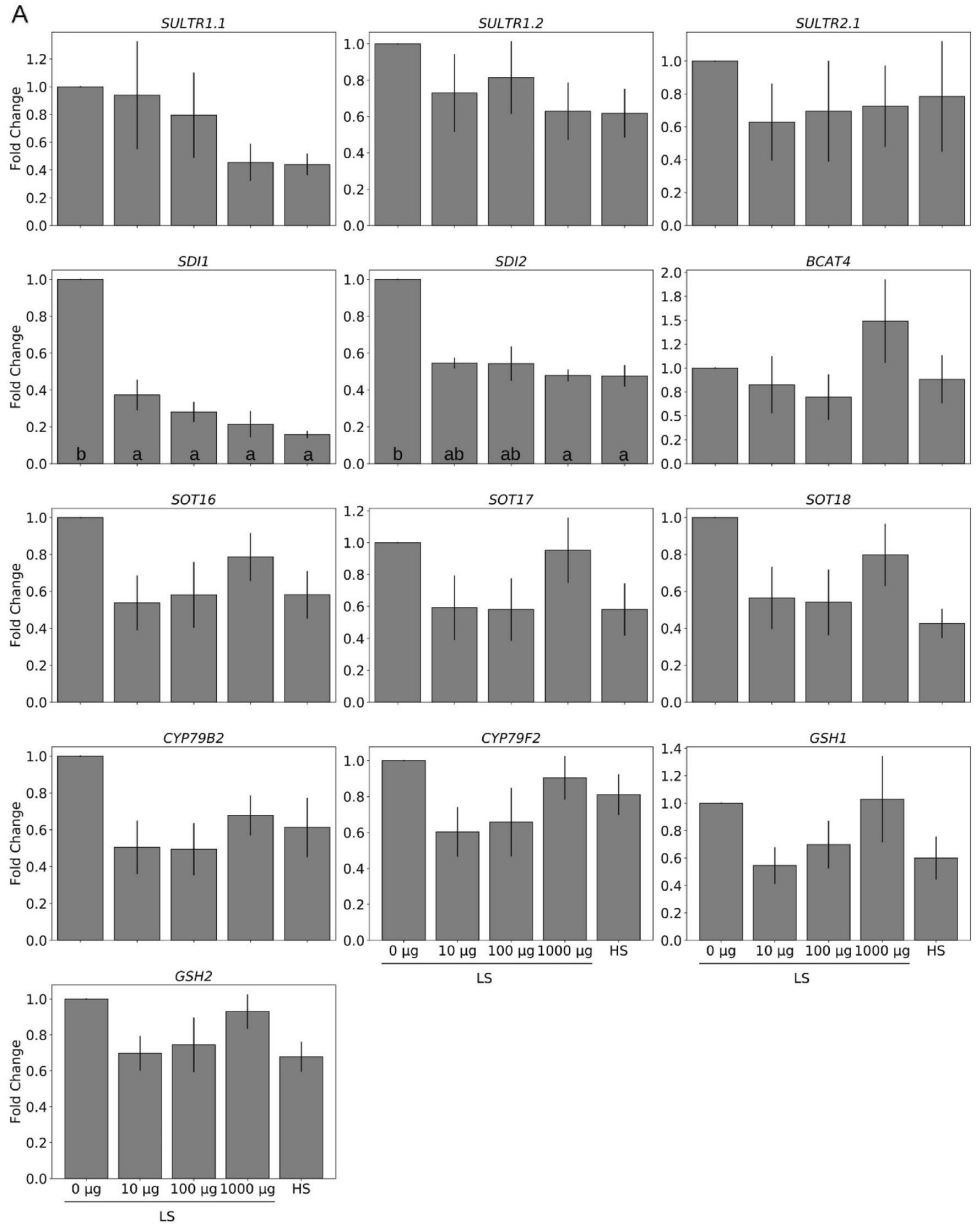

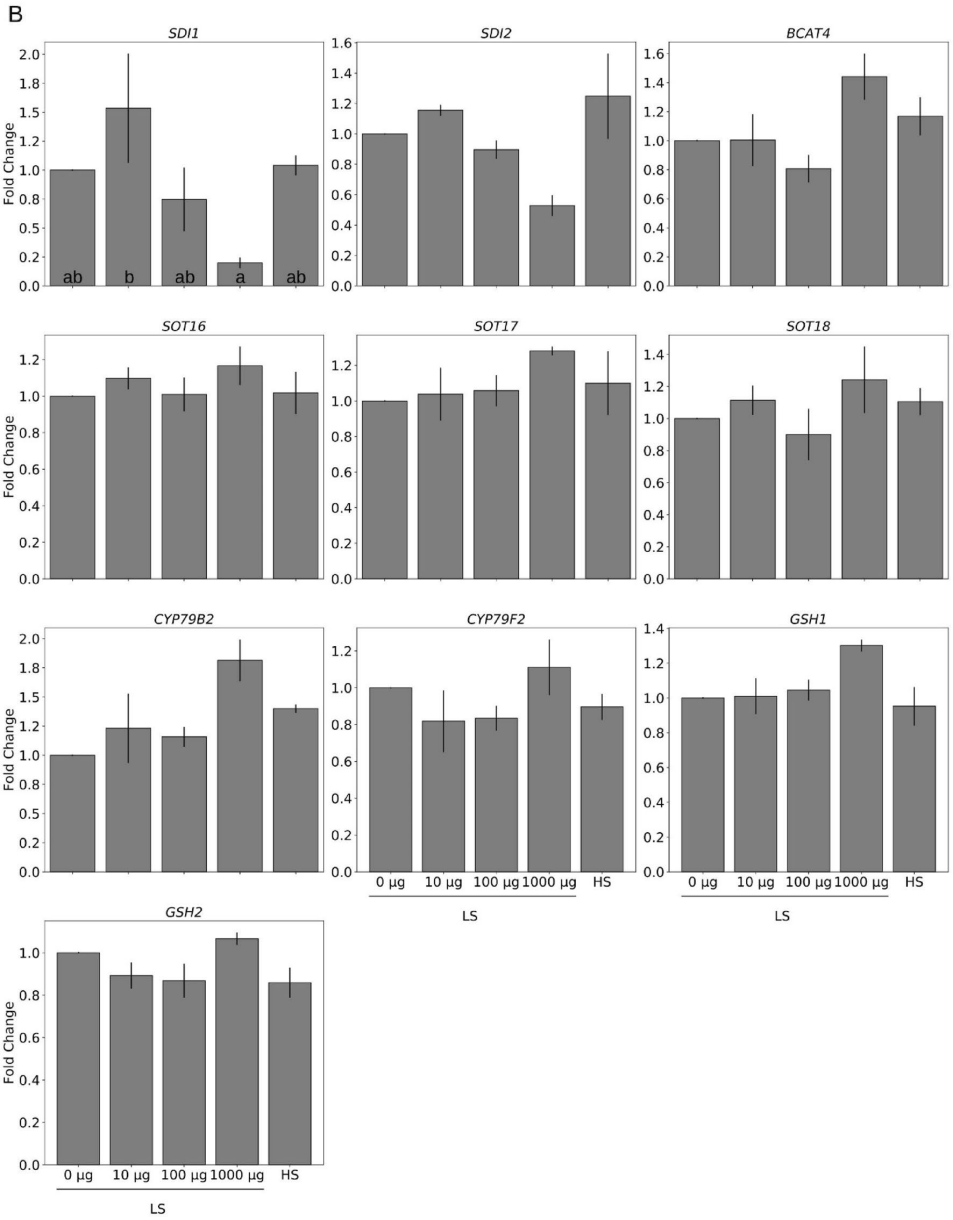
TMTM reduces plant response towards sulfur deficiency. Gene expression was analyzed 2 days (**A**) and 7 days (**B**) after TMTM application. Values were normalized to seedlings grown on low sulfate (LS) MGRL medium without TMTM (0 µg), and expressed as fold change. RNA from each treatment was extracted from total seedlings (combining root and shoot). Error bars represent SEs from 3 biological replicates, each with 8 seedlings. Statistical significance was conducted on dCq values, determined by Duncan’s multiple range test with p-value < 0.05, and indicated with lower-case alphabets.

To test whether TMTM serves as sulfur source and affects the sulfur homeostasis of *Arabidopsis* seedlings, we tested the effect of the volatile on the expression of sulfur-responsive genes and the sulfur metabolite dynamics. Under sulfur limitation conditions, expression of sulfur transporters *SULTR1;1*, *SULTR1;2* and *SULTR2;1* was upregulated. Two days after exposure to the volatile, we observed a gradual decrease of their transcript levels and the effect increased with increasing TMTM amounts. Furthermore, the expression of the GSL repressor genes *SDI1* and *SDI2* was significantly down-regulated by TMTM, again in a dosage-dependent manner (Fig. 5A). We further examined the expression of genes involved in the GSL and GSH metabolisms (i.e., *BRANCHED-CHAIN AMINOTRANSFERASE4, BCAT4; SULFOTRANSFERASE, SOTs; GLUTAMATE-CYSTEINE LIGASE, GSH1; GLUTATHIONE SYNTHETASE, GSH2;* two *CYTOCHROME P450, CYP79B2 and CYP79F2*). With 10 and 100 µg TMTM, their expression levels were similar to those in seedlings grown on HS medium, and with 1000 µg TMTM, their expression levels increased slightly.

Seven days after volatile application, *SDI1* was significantly up-regulated with 10 µg TMTM, while with 100 or 1000 µg TMTM, both *SDI1* and *SDI2* remained down-regulated compared to LS without TMTM (Fig. 5B). The expression levels of the GSL metabolism genes *CYP79B2* and *SOTs* increased to the levels in seedlings grown on LS without TMTM, and with 1000 µg TMTM, they showed the highest expression.

In conclusion, after the application of 100 µg TMTM to LS-exposed seedlings, expression of the examined genes is similar to that of the seedlings grown on HS medium, and this is observed from the second to the 7^th^ day. We propose that low doses of TMTM (10 and 100 µg) diminish sulfur stress by adjusting the expression of the analyzed genes to the expression levels found under HS conditions. Upregulation of *SDI1* and *SDI2* in LS-grown seedlings exposed to 10 µg TMTM for 7 days indicates that this doses is too low to repress the sulfur-deficiency response after a longer time span.

### TMTM maintained GSH and GSL levels under sulfur deficiency

Cysteine is the first metabolite synthesized during sulfur assimilation, while GSH and GSLs comprise large portions of the total sulfur pool. Under sulfur deficiency, these metabolites are broken down, and the sulfur is recycled for primary growth^4,47^. We measured the GSH and GSLs level to investigate whether TMTM influences the plant sulfur homeostasis at this level.

**Figure 6:**
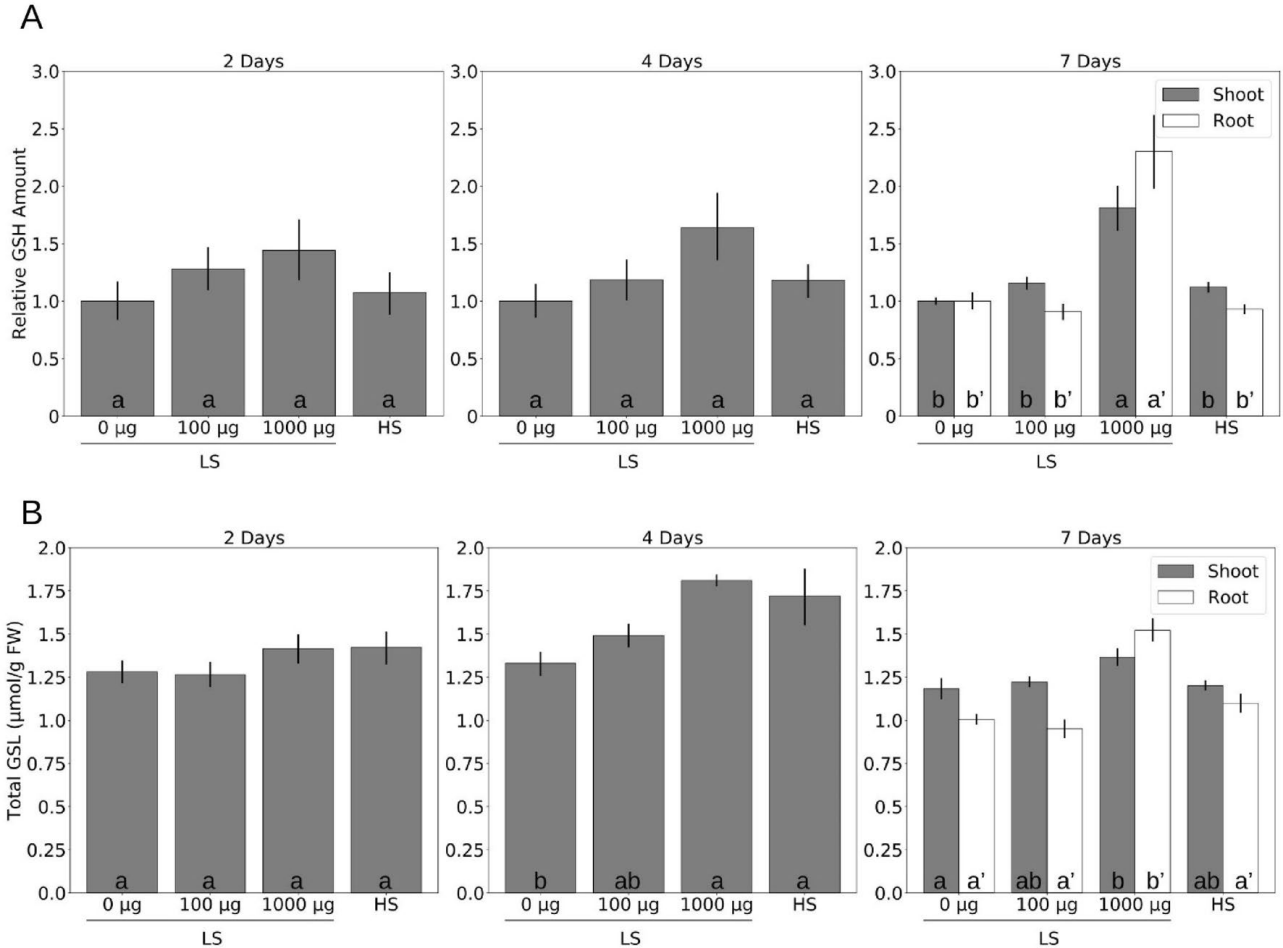
TMTM maintains sulfur-containing metabolites under sulfur deficiency. (**A**) Relative glutathione (GSH) level and (**B**) total glucosinolate (GSL) level in seedlings grown on low sulfate (LS) MGRL medium with addition of TMTM (0, 100 and 1000 µg) and seedlings grown on high sulfate (HS) MGRL medium 2, 4 and 7 days after treatment. Error bars represent SEs from at least 5 biological replicates, each with 8 seedlings. Statistical significance was determined by Duncan’s multiple range test with p-value < 0.05, and indicated with lower-case alphabets.

Two, four and seven days after application of 1000 µg TMTM, the GSH level was significantly increased compared to the untreated control. Even 100 µg TMTM stimulated the GSH level, which was similar to that found in seedlings grown on HS (Fig. 6A).

A similar pattern was observed for the total GSL levels. After 2 days, the GSLs slightly increased with increasing TMTM concentrations (Fig. 6B). The effect broadened after 4 days. On LS without TMTM, total GSL level was significantly lowered compared to the rest of the treatments, indicating the breakdown of GSLs under sulfur limitation (Fig. 6B). Similar to the results obtained for GSH, application of 100 µg TMTM maintained the total GSL level at the same level found in seedlings grown on HS medium without the volatile after 7 days (Fig. 6B). We conclude that 100 µg TMTM established sulfur homeostasis in LS-grown seedlings which is comparable to the conditions in seedlings grown on HS. Furthermore, incorporation of TMTM can be observed in seedlings treated with 1000 µg TMTM, since they showed significantly higher amounts of GSH and GSLs than the unexposed controls.

### OASTLs do not incorporate sulfur from TMTM directly into cysteine

**Figure 7:**
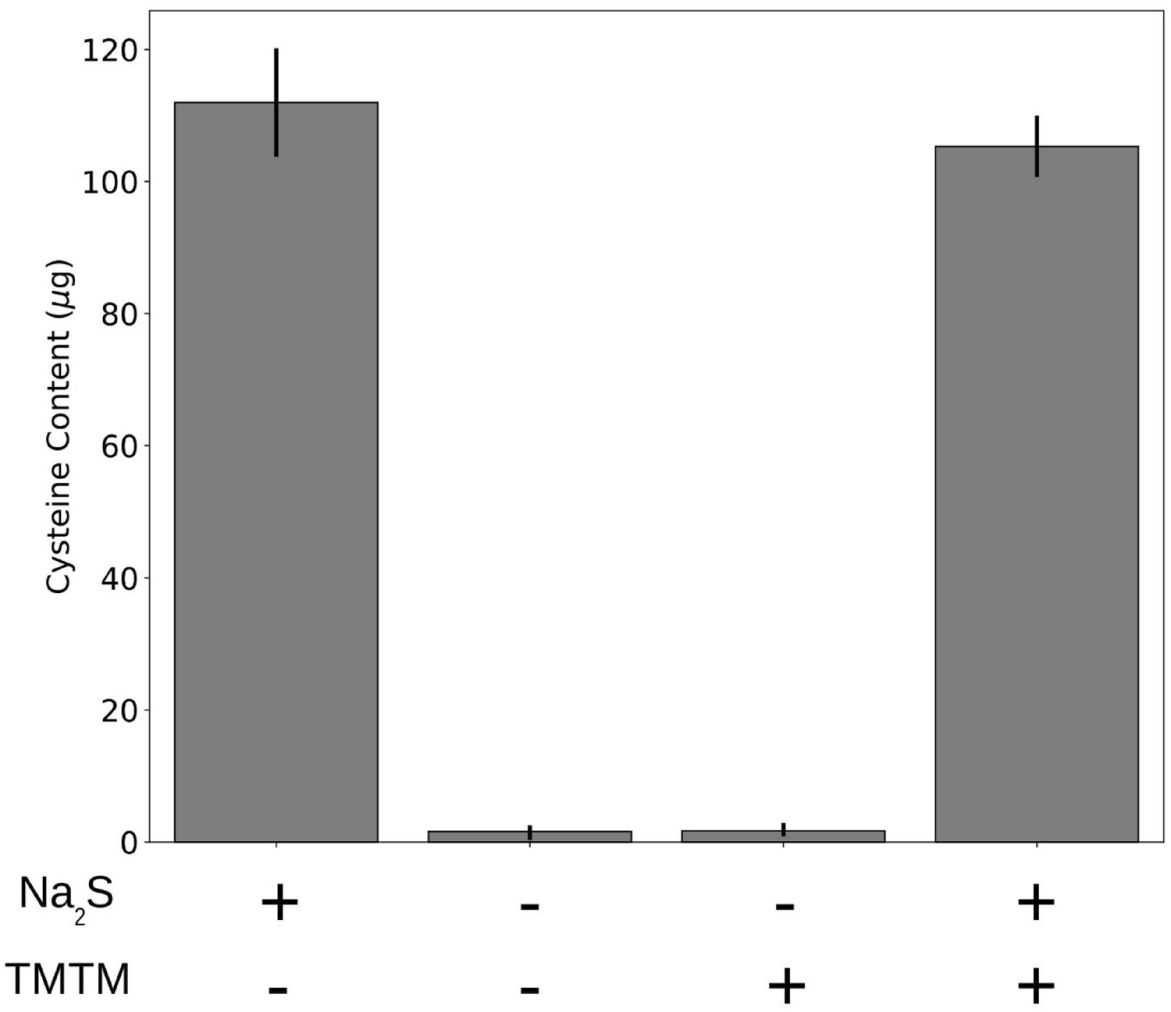
Incorporation of TMTM requires more than OASTLs. Cysteine biosynthesis was monitored in 4 parallel samples. In each sample, either Na_2_S, water, TMTM or both Na_2_S and TMTM was added as substrate for OASTLs. Error bars represent SEs from 3 independent measurement using total protein extract from 3 different biological replicates.

Sulfate is normally reduced to sulfide, a substrate for OASTLs to form cysteine. Cysteine is further converted to GSH, methionine or other sulfur-containing metabolites. TMTM is an organosulfide, containing 3 sulfide groups. We tested if plants can synthesize cysteine using TMTM as substrate. An OASTL activity assay was conducted by incubating total protein extract from wild-type *A. thaliana* (ecotype *Col-0*) leaves with OAS and either Na_2_S or TMTM as substrate. Cysteine production was only observed when Na_2_S was used as substrate (Fig. 7). In another experimental setup, total protein extract, OAS, Na_2_S and TMTM were incubated in the same reaction tube. Also under this condition, cysteine was produced, which indicates that OASTL activity was not hindered by TMTM. We conclude that TMTM is not a direct substrate for sulfur incorporation into cysteine by OASTLs under our experimental conditions (Fig. 7).

## Discussion

In this study, we identified a fungal volatile, TMTM, as the main component in the headspace of the beneficial fungus *M. hyalina*. Application of TMTM participated in maintaining the sulfur homeostasis in *Arabidopsis* seedlings under sulfur deficiency. At low concentrations (10–100 µg), TMTM compensated sulfur-limitation responses of the seedlings: the volatile restored growth and root development which were inhibited under sulfur-limiting conditions, by restricting the up-regulation of sulfur deficiency marker genes (*SULTRs*, *SDI1* and *SDI2*), or the breakdown of GSLs and the accumulation of GSH. On medium with HS, these TMTM effects were not significant. TMTM shifted the measured parameters in LS plants to those found in seedlings grown on HS medium without TMTM application. Higher concentration induced toxic or inhibitory effects, altered the sulfur homeostasis, and restricted plant growth. However, TMTM was not directly incorporated into cysteine by OASTLs, and is not inhibiting their function. This suggests that cysteine might not be a direct product of TMTM incorporation, or TMTM must be processed before its sulfur atoms can be incorporated into cysteine.

Plants reduce the CO_2_ concentration in closed systems which has to be considered in experimental designs with volatiles^48,49^. In preliminary experiments, we co-cultivated *Arabidopsis* seedlings with 5 different *Mortierella* strains with comparable growth rates and metabolite features. Since the three fungi with distinctive garlic-like smells (*M. hyalina*, *M. alpina*, *M. turficola*) induced a stronger growth promotion compared to two non-smelling strains (*M. vinacea*, *M. longicollis*; Fig. S1), we hypothesized that the sulfur-containing volatiles from the fungi might be involved in the growth regulation (Fig. S1). The major volatile in the headspace of one of these fungi, *M. hyalina*, was TMTM, and its abundance prompted us to investigate it in this study. The stronger growth of seedlings which are growing in the presence of the fungus compared to those treated with TMTM demonstrates that the investigated volatile is not the only factor involved in the growth promoting effect. Nevertheless, the stabilizing effect of TMTM on the sulfur homeostasis allows better plant performance under sulfur stress.

Plants respond to sulfur limitation in various ways. The first response is the up-regulation of sulfate transporters (*SULTRs*) to increase sulfate uptake from root^24–28^. On the other hand, genes for GSL biosynthesis (e.g., *BCAT4*, *CYP79B2* and *CYP79F2*) are down-regulated, while those repressing GSL biosynthesis (*SDI1* and *SDI2*) are up-regulated. These responses help plants to remobilize sulfur to sustain growth^50–52^. Among the inspected genes, the sulfur starvation genes *SULTR1;1*, *SULTR1;2*, *SULTR2;1* and both *SDI1* and *SDI2* were down-regulated in a TMTM dose-dependent manner in plants which suffer from sulfur limitation (Fig. 5). Since the sulfur in TMTM can be incorporated into the plant material (Fig. 2), the expression of the above-mentioned genes and those involved in GSL and GSH metabolism is similar to seedlings grown under HS condition without TMTM. Moreover, excessive TMTM results in the up-regulation of these genes, indicating that these plants are actively moving excess sulfur to secondary metabolites. This is in accordance with a recent study by^47^, showing a retrograde sulfur flow from glucosinolates to cysteine in *Arabidopsis.* Interestingly, the mRNA levels for GSL and GSH metabolism genes were higher in seedlings after 2 days on LS medium without TMTM compared to seedlings grown on HS medium (Fig. 6). This might be caused by higher sulfate influx from the medium due to up-regulation of *SULTRs*. The plants actively metabolize the assimilated sulfate into various metabolites and utilize this as a storage to sustain growth under sulfur limitation.

The effect is also observed at the metabolic level. Under sulfur limitation, the GSH and GSL levels decreased. However, 100 µg TMTM maintained the levels under sulfur starvation conditions (Fig. 6). Again, besides maintaining sulfur homeostasis, excess sulfur from the high TMTM dose is largely metabolized into secondary metabolites.

Imbalances in the sulfur pool have severe consequences for plant growth and yield^53–55^. Under LS, biomass production and root growth were significantly reduced (Figs. 3 and 4). This could be restored by the application of TMTM in low doses. We propose that TMTM maintains the sulfur homeostasis and allows root growth which is comparable to that under HS conditions (Fig. 4). Furthermore, it appears that excess TMTM tilts the sulfur homeostasis and shifts the sulfur towards the secondary metabolite pool, resulting in reduced plant biomass production and root growth (Figs. 3 and 4).

Growth regulation by TMTM via sulfur homeostasis is further supported by the response of WT and *slim1* seedlings to high TMTM dose. 1000 µg TMTM inhibited root growth in WT seedlings, but not in *slim1* (Fig. 4). Apparently, lower doses of TMTM stimulated root growth in LS because the volatile influences the sulfur homeostasis. As a result, the root growth was comparable to seedling´s growth on HS without the volatile. However, the high dose (1000 µg) of TMTM could provide too much sulfur to the LS-grown WT seedlings, which may result in the activation of stress responses and ultimately growth retardation. On the other hand, because *slim1* could not mobilize sulfur from its secondary metabolites^31^, these seedlings showed a higher tolerance to excess TMTM. The different response of the two genotypes to excess TMTM is consistent with the idea that TMTM induces changes in the sulfur homeostasis which influences root growth.

It is known that plants are able to assimilate gaseous sulfur compounds, such as SO_2_ and H_2_S^21,56,57^. They are also able to assimilate other sulfur-containing organic volatiles produced by microbes. One example is dimethyl disulfide^18^. Nevertheless, how organosulfides are metabolized inside the plant remains unknown.

Diallyl disulfide (DADS), a volatile from garlic, is perhaps the best studied organosulfide due to its anticancer ability^58–61^. It increases GSH levels and regulates antioxidant enzyme activity, leading to reduced oxidative stress in animal models^62–64^. In plants, DADS also affects the expression of sulfur metabolism genes^65–67^. Metabolism of DADS and other organosulfides generates H_2_S^68–71^. Studies on how DADS and other organosulfides are metabolized suggest the involvement of GSH and cysteine^69–71^. The reaction between DADS and GSH produces S-allyl GSH and a short-lived intermediate allyl perthio through α-carbon nucleophilic substitution. The allyl perthio reacts with a second GSH, resulting in the release of H_2_S and S-allyl GSH disulfide^71^.

We found that 1000 µg TMTM increased both the GSH and GSL levels, and the GSH level responded faster to the volatile treatment (Fig. 6). A possible explanation could be that incorporation of sulfur from TMTM into the plant metabolism is connected to GSH. We tested if TMTM can be a direct substrate for OASTLs and found that this is not the case (Fig. 7). Therefore, unlike sulfate assimilation, TMTM might first interfere with the GSH/GSSG system. This might lead to the cleavage of the C–S bonds and sulfur incorporation into plant. A detailed metabolome analysis of early sulfur-containing compounds after TMTM treatment might elucidate the early steps in the role of this novel volatile.

## Supplementary Information

Supplementary Information.

## References

[1] Raven JA, Evans MCW, Korb RE (1999). The role of trace metals in photosynthetic electron transport in O_2_-evolving organisms. Photosynth. Res..

[2] Bakhtiari M, Rasmann S (2020). Variation in below-to aboveground systemic induction of glucosinolates mediates plant fitness consequences under herbivore attack. J. Chem. Ecol..

[3] Ting H-M (2020). The role of a glucosinolate-derived nitrile in plant immune responses. Front. Plant Sci..

[4] Falk KL, Tokuhisa JG, Gershenzon J (2007). The effect of sulfur nutrition on plant glucosinolate content: Physiology and molecular mechanisms. Plant Biol..

[5] Aghajanzadeh T, Hawkesford MJ, De Kok LJ (2014). The significance of glucosinolates for sulfur storage in Brassicaceae seedlings. Front. Plant Sci..

[6] Lipman JG, Mclean HC, Lint HC (1916). Sulfur oxidation in soils and its effect on the availability of mineral phosphates. Soil Sci..

[7] Beijerinck MW (1904). Phenomenes de reduction produits par les microbes. Arch. Néerlandaises Sci. Exactes Nat. (Sect. 2).

[8] Waksman SA, Joffe JS (1922). Microörganisms concerned in the oxidation of sulfur in the soil: II. *Thiobacillus thiooxidans*, a new sulfur-oxidizing organism isolated from the soil. J. Bacteriol..

[9] Deng SP, Tabatabai MA (1997). Effect of tillage and residue management on enzyme activities in soils: III. Phosphatases and arylsulfatase. Biol. Fertil. Soils.

[10] Kertesz MA (2000). Riding the sulfur cycle-metabolism of sulfonates and sulfate esters in gram-negative bacteria. FEMS Microbiol. Rev..

[11] Fitzgerald JW (1976). Sulfate ester formation and hydrolysis: A potentially important yet often ignored aspect of the sulfur cycle of aerobic soils. Bacteriol. Rev..

[12] Marzluf GA (1997). Molecular genetics of sulfur assimilation in filamentous fungi and yeast. Annu. Rev. Microbiol..

[13] Omar SA, Abd-Alla MH (2000). Physiological aspects of fungi isolated from root nodules of faba bean (*Vicia faba* L.). Microbiol. Res..

[14] Baum C, Hrynkiewicz K (2006). Clonal and seasonal shifts in communities of saprotrophic microfungi and soil enzyme activities in the mycorrhizosphere of *Salix* spp.. J. Plant Nutr. Soil Sci..

[15] Gray LE, Gerdemann JW (1973). Uptake of sulphur-35 by vesicular-arbuscular mycorrhizae. Plant Soil.

[16] Cavagnaro TR (2006). Arbuscular mycorrhizas, microbial communities, nutrient availability, and soil aggregates in organic tomato production. Plant Soil.

[17] Giovannetti M, Tolosano M, Volpe V, Kopriva S, Bonfante P (2014). Identification and functional characterization of a sulfate transporter induced by both sulfur starvation and mycorrhiza formation in *Lotus japonicus*. New Phytol..

[18] Meldau DG (2013). Dimethyl disulfide produced by the naturally associated bacterium Bacillus sp B55 promotes *Nicotiana attenuata* growth by enhancing sulfur nutrition. Plant Cell.

[19] Kai M (2010). *Serratia odorifera*: Analysis of volatile emission and biological impact of volatile compounds on *Arabidopsis thaliana*. Appl. Microbiol. Biotechnol..

[20] Dickschat JS (2017). Fungal volatiles—A survey from edible mushrooms to moulds. Nat. Prod. Rep..

[21] Randewig D (2012). Sulfite oxidase controls sulfur metabolism under SO_2_ exposure in *Arabidopsis thaliana*. Plant Cell Environ..

[22] Pfanz H, Martinoia E, Lange O-L, Heber U (1987). Mesophyll resistances to SO_2_ fluxes into leaves. Plant Physiol..

[23] Rennenberg, H. & Polle, A. Metabolic consequences of atmospheric sulphur influx into plants. in *Plant Responses to the Gaseous Environment: Molecular, metabolic and physiological aspects* (eds. Alscher, R. G. & Wellburn, A. R.). 165–180. 10.1007/978-94-011-1294-9_9 (Springer, 1994).

[24] Takahashi H, Kopriva S, Giordano M, Saito K, Hell R (2011). Sulfur assimilation in photosynthetic organisms: Molecular functions and regulations of transporters and assimilatory enzymes. Annu. Rev. Plant Biol..

[25] Shibagaki N (2002). Selenate-resistant mutants of *Arabidopsis thaliana* identify Sultr1;2, a sulfate transporter required for efficient transport of sulfate into roots. Plant J..

[26] Yoshimoto N, Takahashi H, Smith FW, Yamaya T, Saito K (2002). Two distinct high-affinity sulfate transporters with different inducibilities mediate uptake of sulfate in Arabidopsis roots. Plant J..

[27] Kataoka T, Hayashi N, Yamaya T, Takahashi H (2004). Root-to-shoot transport of sulfate in Arabidopsis: Evidence for the role of Sultr3;5 as a component of low-affinity sulfate transport system in the root vasculature. Plant Physiol..

[28] Takahashi H (1997). Regulation of sulfur assimilation in higher plants: A sulfate transporter induced in sulfate-starved roots plays a central role in *Arabidopsis thaliana*. Proc. Natl. Acad. Sci..

[29] Mugford SG, Lee B-R, Koprivova A, Matthewman C, Kopriva S (2011). Control of sulfur partitioning between primary and secondary metabolism: Sulfur partitioning. Plant J..

[30] Mugford SG (2009). Disruption of adenosine-5′-phosphosulfate kinase in *Arabidopsis* reduces levels of sulfated secondary metabolites. Plant Cell.

[31] Maruyama-Nakashita A, Nakamura Y, Tohge T, Saito K, Takahashi H (2006). Arabidopsis SLIM1 is a central transcriptional regulator of plant sulfur response and metabolism. Plant Cell.

[32] Aarabi F (2016). Sulfur deficiency-induced repressor proteins optimize glucosinolate biosynthesis in plants. Sci. Adv..

[33] Hirai MY (2007). Omics-based identification of Arabidopsis Myb transcription factors regulating aliphatic glucosinolate biosynthesis. Proc. Natl. Acad. Sci. USA.

[34] Gigolashvili T, Yatusevich R, Berger B, Müller C, Flügge U-I (2007). The R2R3-MYB transcription factor HAG1/MYB28 is a regulator of methionine-derived glucosinolate biosynthesis in *Arabidopsis thaliana*. Plant J..

[35] Johnson JM (2019). the beneficial root-colonizing fungus *Mortierella hyalina* promotes the aerial growth of *Arabidopsis* and activates calcium-dependent responses that restrict *Alternaria brassicae* –induced disease development in roots. Mol. Plant. Microbe Interact..

[36] Murashige T, Skoog F (1962). A revised medium for rapid growth and bio assays with tobacco tissue cultures. Physiol. Plant..

[37] Fujiwara T, Hirai MY, Chino M, Komeda Y, Naito S (1992). Effects of sulfur nutrition on expression of the soybean seed storage protein genes in transgenic petunia. Plant Physiol..

[38] Bains PS, Tewari JP (1987). Purification, chemical characterization and host-specificity of the toxin produced by *Alternaria brassicae*. Physiol. Mol. Plant Pathol..

[39] Hill T, Käfer E (2001). Improved protocols for Aspergillus minimal medium: Trace element and minimal medium salt stock solutions. Fungal Genet. Newsl..

[40] National Institute of Standards and Technology. *NIST/EPA/NIH Mass Spectral & Retention Index Library*. (2014).

[41] Hochmuth D (2010). Massfinder v. 4.21.

[42] Brown PD, Tokuhisa JG, Reichelt M, Gershenzon J (2003). Variation of glucosinolate accumulation among different organs and developmental stages of *Arabidopsis thaliana*. Phytochemistry.

[43] Burow M, Müller R, Gershenzon J, Wittstock U (2006). Altered glucosinolate hydrolysis in genetically engineered *Arabidopsis thaliana* and its influence on the larval development of *Spodoptera littoralis*. J. Chem. Ecol..

[44] Vancompernolle B, Croes K, Angenon G (2016). Optimization of a gas chromatography-mass spectrometry method with methyl chloroformate derivatization for quantification of amino acids in plant tissue. J. Chromatogr. B Anal. Technol. Biomed. Life. Sci..

[45] Schindelin J (2012). Fiji: An open-source platform for biological-image analysis. Nat. Methods.

[46] Lobet G, Pagès L, Draye X (2011). A novel image-analysis toolbox enabling quantitative analysis of root system architecture. Plant Physiol..

[47] Sugiyama R (2021). Retrograde sulfur flow from glucosinolates to cysteine in Arabidopsis thaliana. Proc. Natl. Acad. Sci..

[48] Naznin HA, Kimura M, Miyazawa M, Hyakumachi M (2013). Analysis of volatile organic compounds emitted by plant growth-promoting fungus *Phoma* sp. GS8-3 for growth promotion effects on tobacco. Microbes Environ..

[49] Piechulla B, Lemfack MC, Kai M (2017). Effects of discrete bioactive microbial volatiles on plants and fungi. Plant Cell Environ..

[50] Lewandowska M, Sirko A (2008). Recent advances in understanding plant response to sulfur-deficiency stress. Acta Biochim. Pol..

[51] Frerigmann H, Gigolashvili T (2014). Update on the role of R2R3-MYBs in the regulation of glucosinolates upon sulfur deficiency. Front. Plant Sci..

[52] Borpatragohain P, Rose TJ, King GJ (2016). Fire and brimstone: molecular interactions between sulfur and glucosinolate biosynthesis in model and crop Brassicaceae. Front. Plant Sci..

[53] Zhao F, Hawkesford M, McGrath S (1999). Sulphur assimilation and effects on yield and quality of wheat. J. Cereal Sci..

[54] Lunde C (2008). Sulfur starvation in rice: The effect on photosynthesis, carbohydrate metabolism, and oxidative stress protective pathways. Physiol. Plant..

[55] Jobe TO, Zenzen I, RahimzadehKarvansara P, Kopriva S (2019). Integration of sulfate assimilation with carbon and nitrogen metabolism in transition from C3 to C4 photosynthesis. J. Exp. Bot..

[56] Lee HK (2017). The relationship between SO_2_ exposure and plant physiology: A mini review. Hortic. Environ. Biotechnol..

[57] Ausma T, De Kok LJ (2019). Atmospheric H_2_S: Impact on plant functioning. Front. Plant Sci..

[58] Yi L, Su Q (2013). Molecular mechanisms for the anti-cancer effects of diallyl disulfide. Food Chem. Toxicol. Int. J. Publ. Br. Ind. Biol. Res. Assoc..

[59] Xiong T (2018). Tristetraprolin: A novel target of diallyl disulfide that inhibits the progression of breast cancer. Oncol. Lett..

[60] Agassi SFT, Yeh T-M, Chang C-D, Hsu J-L, Shih W-L (2020). Potentiation of differentiation and apoptosis in a human promyelocytic leukemia cell line by garlic essential oil and its organosulfur compounds. Anticancer Res..

[61] Li Y, Wang Z, Li J, Sang X (2018). Diallyl disulfide suppresses FOXM1-mediated proliferation and invasion in osteosarcoma by upregulating miR-134. J. Cell. Biochem..

[62] Demeule M (2004). Diallyl disulfide, a chemopreventive agent in garlic, induces multidrug resistance-associated protein 2 expression. Biochem. Biophys. Res. Commun..

[63] Hassanein EHM (2021). Diallyl disulfide ameliorates methotrexate-induced nephropathy in rats: Molecular studies and network pharmacology analysis. J. Food Biochem..

[64] Wei X (2021). Acute diallyl disulfide administration prevents and reveres lipopolysaccharide-induced depression-like behaviors in mice via regulating neuroinflammation and oxido-nitrosative stress. Inflammation.

[65] Cheng F, Cheng Z-H, Meng H-W (2016). Transcriptomic insights into the allelopathic effects of the garlic allelochemical diallyl disulfide on tomato roots. Sci. Rep..

[66] Cheng F, Ali M, Liu C, Deng R, Cheng Z (2020). Garlic allelochemical diallyl disulfide alleviates autotoxicity in the root exudates caused by long-term continuous cropping of tomato. J. Agric. Food Chem..

[67] Yang F (2019). Identification and allelopathy of green garlic (*Allium sativum* L.) volatiles on scavenging of cucumber (*Cucumis sativus* L.) reactive oxygen species. Mol. Basel Switz..

[68] Kim TJ, Lee YJ, Ahn YJ, Lee G-J (2019). Characterization of H_2_S releasing properties of various H_2_S donors utilizing microplate cover-based colorimetric assay. Anal. Biochem..

[69] Cai Y-R, Hu C-H (2017). Computational study of H_2_S release in reactions of diallyl polysulfides with thiols. J. Phys. Chem. B.

[70] Bolton SG, Cerda MM, Gilbert AK, Pluth MD (2019). Effects of sulfane sulfur content in benzyl polysulfides on thiol-triggered H2S release and cell proliferation. Free Radic. Biol. Med..

[71] Liang D, Wu H, Wong MW, Huang D (2015). Diallyl trisulfide is a fast H_2_S donor, but diallyl disulfide is a slow one: the reaction pathways and intermediates of glutathione with polysulfides. Org. Lett..

