# Supplementary material for: Tris(methylthio)methane produced by Mortierella hyalina affects sulfur homeostasis in Arabidopsis
Source: Sci Rep. 2022 Aug 20;12:14202. doi: 10.1038/s41598-022-16827-7 (PMC9392766; doi:10.1038/s41598-022-16827-7)
Supplement: Supplementary file 1 — Supplementary Information. [file 41598_2022_16827_MOESM1_ESM.pdf]

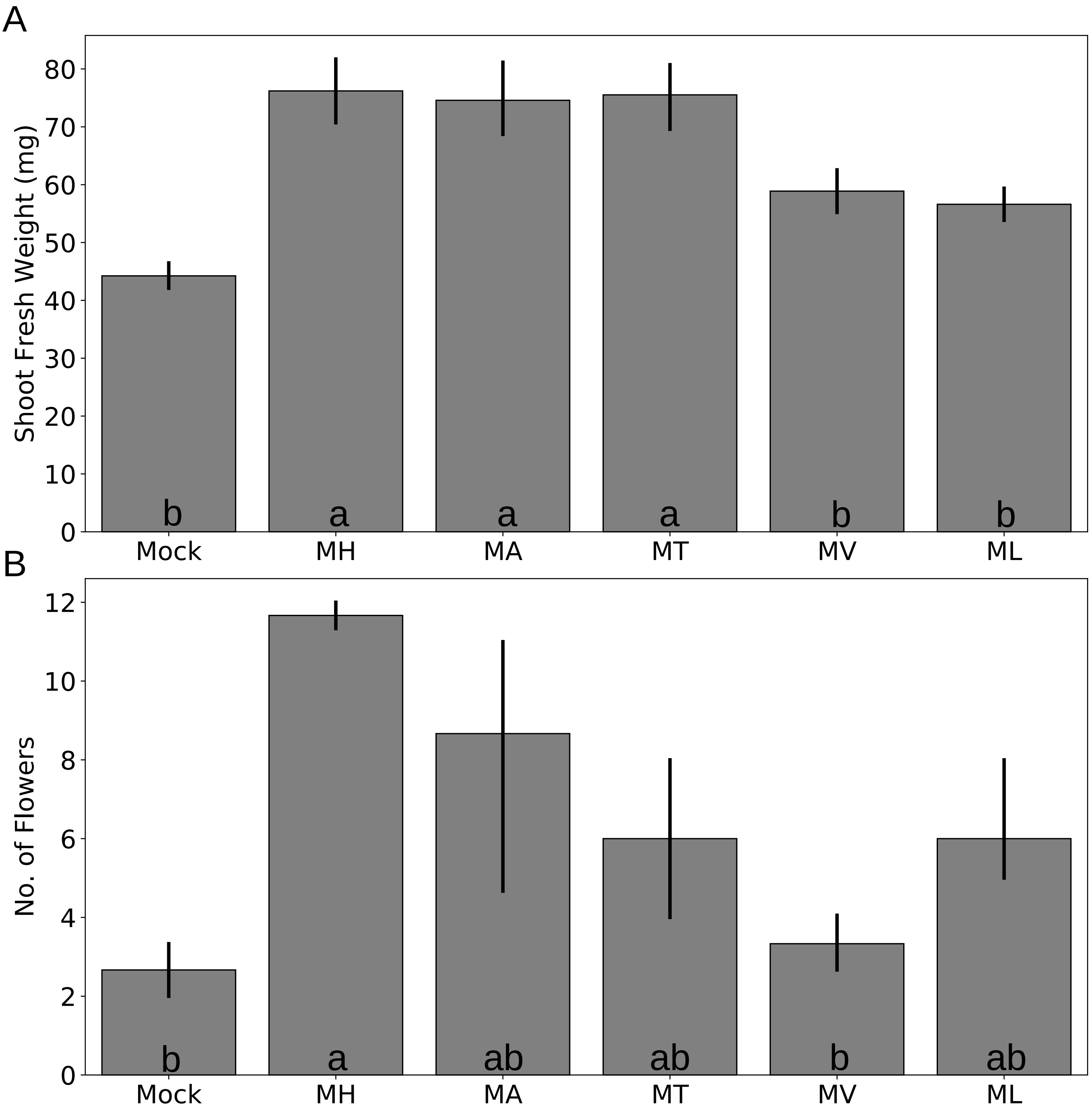

Figure S1. Fungal volatiles from *M. hyalina* promote *Arabidopsis* growth. Shown are (A) Shoot fresh weight and (B) number of flower buds. Mock, without fungus. MH, *Mortierella hyalina*. MA, *Mortierella alpina*. MT, *Mortierella turficola*. ML, *Mortierella longicollis*. MV, *Mortierella vinacea.* Error bars represent SEs from at least 3 independent biological replicates, each with 21 seedlings. Statistical significance was determined by Duncan's multiple range test with p-value < 0.05, and indicated with lower-case alphabets.

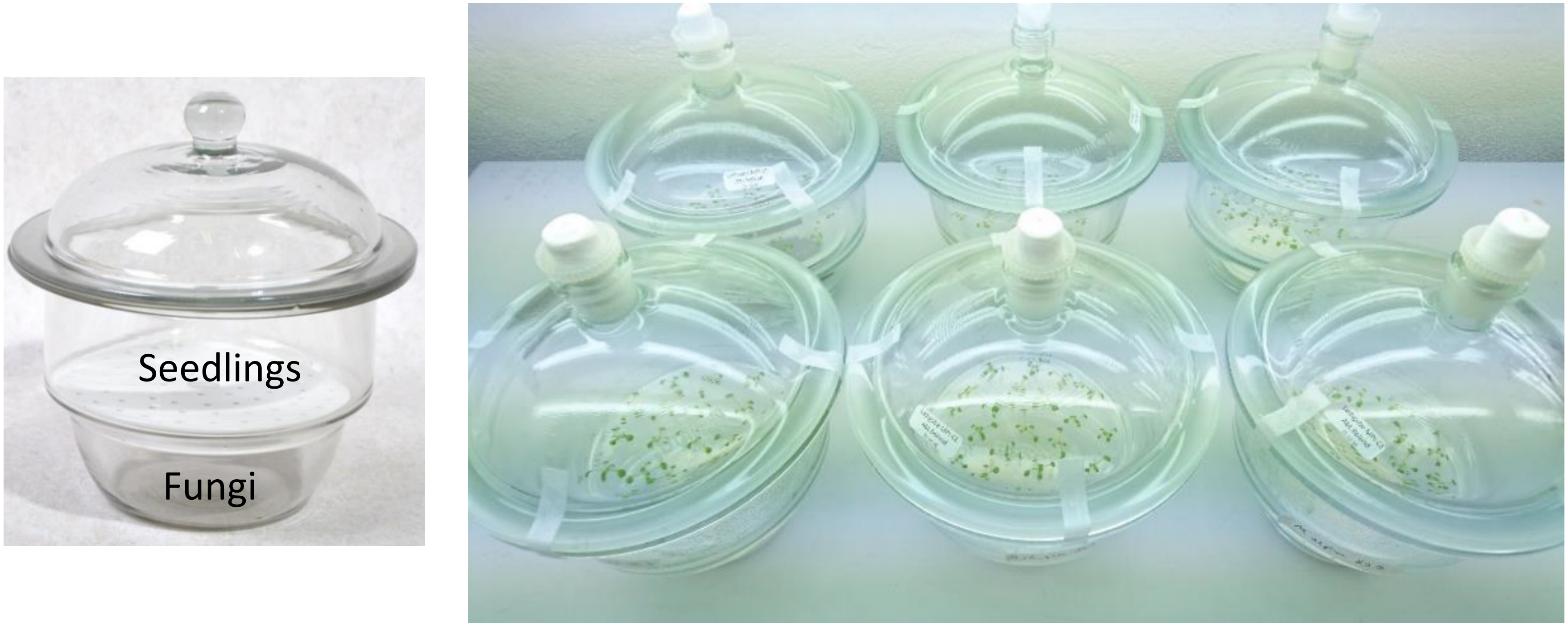

Figure S2. Experimental set-up for fungi-*Arabidopsis* co-cultivation in a desiccator with shared headspace.

Figure S3. Structures of the different glucosinolates (GSLs). Left: core structure of GSL. Right: side-chain (R) of different GSL species.

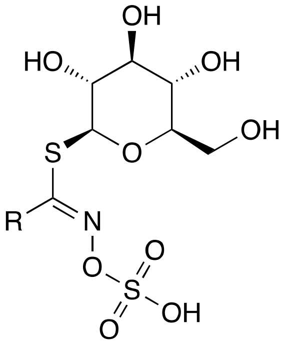

Name	R
3-Indolylmethyl glucosinolate (I3M)	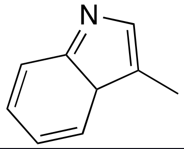
(4-Methoxy-3-indolylmethyl) glucosinolate (4MOI3M)	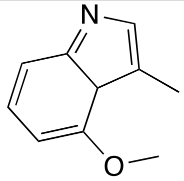
4-Methylthiobutyl glucosinolate (4MTB)	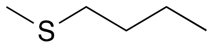
4-Methylsulfinylbutyl glucosinolate (4MSOB)	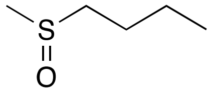
8-Methylsulfinyloctyl glucosinolate (8MSOO)	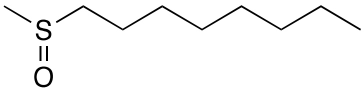

Figure S4. Structure of glutathione (GSH).

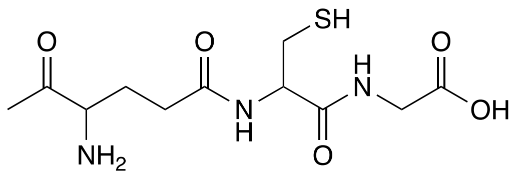

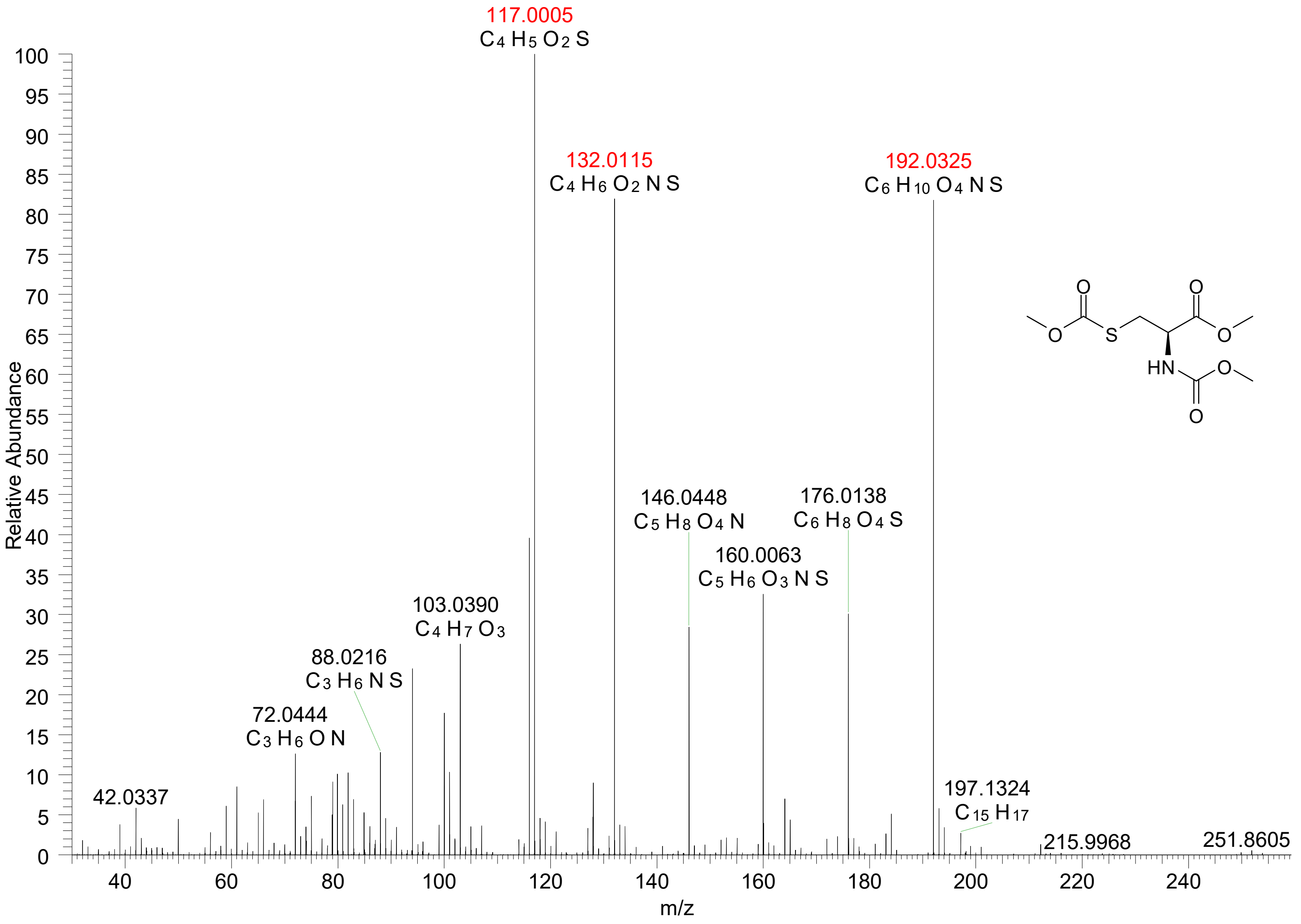

Figure S5. HR-MS spectrum for cysteine derivative. The structure of derived cysteine is shown on the right. Fragments used for the calculation of $^{34}S/^{32}S$ ratio are marked in red.

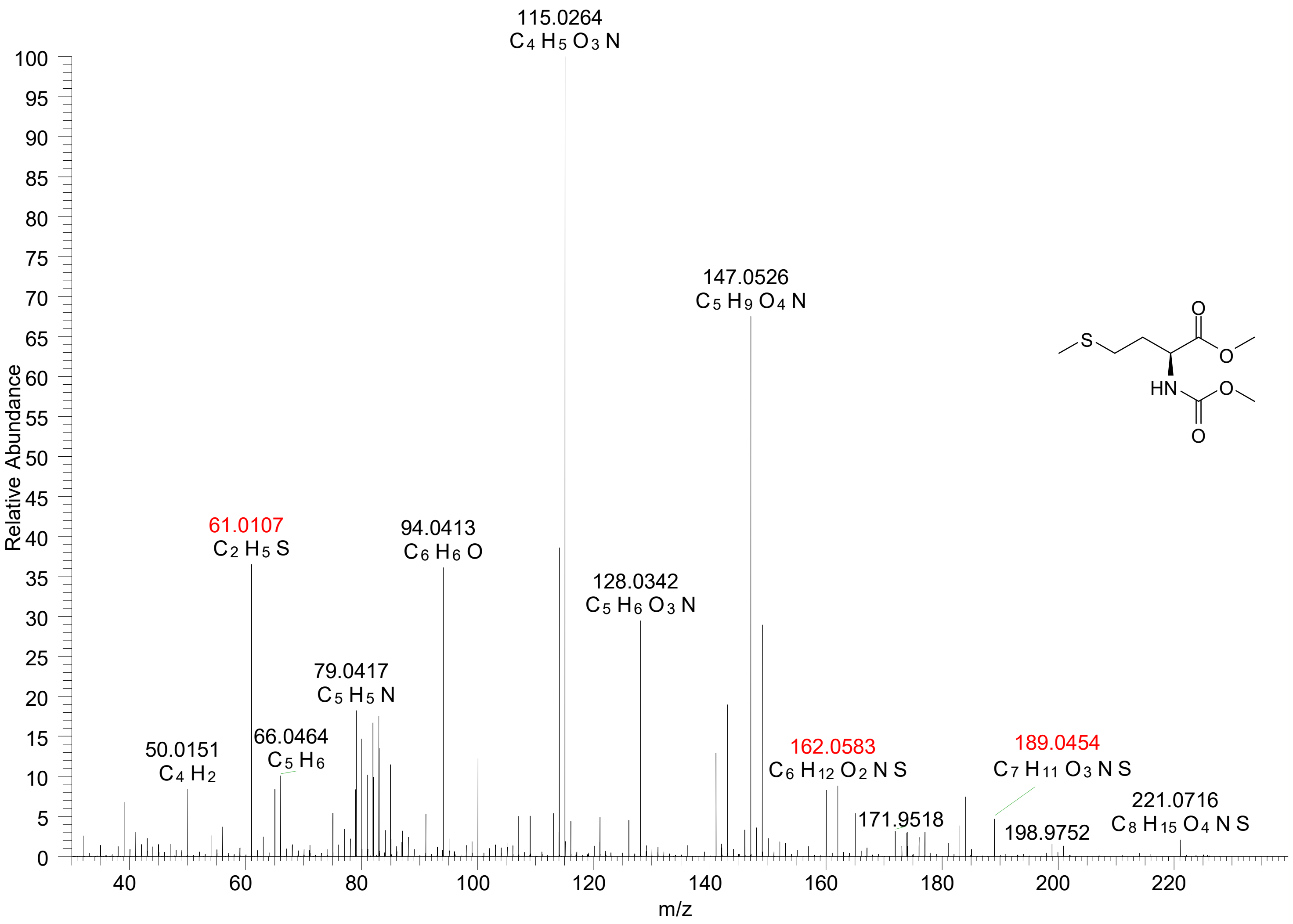

Figure S6. HR-MS spectrum for methionine derivative. The structure of derived methionine is shown on the right. Fragments used for the calculation of $^{34}S/^{32}S$ ratio are marked in red.

Table S1. Primers used in this study.

Gene	Accession Number	Sequence (5‘-3‘)
RPS18B	AT1G34030	RPS-qF-GTCTCCAATGCCCTTGACAT
		RPS-qR-TCTTTCCTCTGCGACCAGTT
SULTR1.1	AT4G08620	SULTR1.1-qF-GCCATCACAATCGCTCTCCAA
		SULTR1.1-qR-TTGCCAATTCCACCCATGC
SULTR1.2	AT1G78000	SULTR1.2-qF-GGATCCAGAGATGGCTACATGA
		SULTR1.2-qR-TCGATGTCCGTAACAGGTGAC
SULTR2.1	AT5G10180	SULTR2.1-qF-ATTGTTGCTCTAACCGAGGCGATT
		SULTR2.1-qR-TGTACCCTTTTATTCCGGCGAACG
SDI1	AT5G48850	SDI1-qF-TCAGAGCCAAACATGCTCAGTT
		SDI1-qR-ACAACAGCCATGTCCTTGAGG
SDI2	AT1G04770	SDI2-qF-CGAGCGAAGCATGTTCAGTTG
		SDI2-qR-CGTCAATGGCTTCTTCAGCTCT
BCAT4	AT3G19710	BCAT4-qF-CAGAAGATGGTCGGATTCTGCTA
		BCAT4-qR-GGCAAAAGCTGTGAAGGTGGT
SOT16	AT1G74100	SOT16-qF-CGAAGTCGTCGAACTCACAGAGTT
		SOT16-qR-AAAGACCTTCGAGGAGACATTCTTG
SOT17	AT1G18590	SOT17-qF-GGAATCCAAAACCATAAACGACG
		SOT17-qR-CGGATCTTTTGGTCTCCAGCC
SOT18	AT1G74090	SOT18-qF-CCCTACCGAGTCACGACGAGA
		SOT18-qR-GGTAGCCACCAGTAACCACCATACT
GSH1	AT4G23100	GSH1-qF-GACAGACACTGACAAGGACCGC
		GSH1-qR-CAACATACTGCTCAAACCCAAAAGA
GSH2	AT5G27380	GSH2-qF-TTGGAGTACAGTAACCCAAGAGCGGTAGT
		GSH2-qR-CATCCTCTTGTACACTCCCTTCTTTTTCGACTT
CYP79B2	AT4G39950	CYP79B2-qF-AAATCAAACCCACCATTAAGGAG
		CYP79B2-qR-GAGAATCTCCGGTTTGTTCAC
CYP79F2	AT1G16400	CYP79F2-qF-CCCATAATAGACGAGAGGGTCGAAA
		CYP79F2-qR-CGATCGCTGCTATACAAAATTCG

Table S2. m/z values of extracted ion chromatogram traces of isotopologues of glucosinolates and glutathione for LC-ESI-Q-ToF-MS analysis for ^{34}S incorporation. 4MSOB GSL, 4-methylsulfinylbutyl glucosinolate; 4MTB GSL, 4-methylthiobutyl glucosinolate; 8MSOO GSL, 8-methylsulfinyloctyl glucosinolate; I3M GSL, indol-3-yl-methyl glucosinolate; 4MOI3M GSL, 4-methoxyindol-3-yl-methyl glucosinolate; GSH, glutathione (reduced form).

Isotopologue	Ionization Mode	m/z
**4MSOB GSL $[M-H]^-$**	neg	436.0411±0.002
**4MSOB GSL isotopologue+1**	neg	437.0446±0.002
**4MSOB GSL isotopologue+2**	neg	438.0379±0.002
**4MSOB GSL isotopologue+3**	neg	439.0422±0.002
**4MSOB GSL isotopologue+4**	neg	440.0348±0.002
**4MTB GSL $[M-H]^-$**	neg	420.0460±0.002
**4MTB GSL isotopologue+1**	neg	421.0490±0.002
**4MTB GSL isotopologue+2**	neg	422.0434±0.002
**4MTB GSL isotopologue+3**	neg	423.0484±0.002
**8MSOO GSL $[M-H]^-$**	neg	492.1038±0.002
**8MSOO GSL isotopologue+1**	neg	493.1068±0.002
**8MSOO GSL isotopologue+2**	neg	494.1017±0.002
**8MSOO GSL isotopologue+3**	neg	495.1020±0.002
**I3M GSL $[M-H]^-$**	neg	447.0541±0.002
**I3M GSL isotopologue+1**	neg	448.0566±0.002
**I3M GSL isotopologue+2**	neg	449.0512±0.002
**I3M GSL isotopologue+3**	neg	450.0558±0.002
**4MOI3M GSL $[M-H]^-$**	neg	477.0646±0.002
**4MOI3M GSL isotopologue+1**	neg	478.0676±0.002
**4MOI3M GSL isotopologue+2**	neg	479.0615±0.002
**4MOI3M GSL isotopologue+3**	neg	480.0669±0.002
**GSH $[M+H]^+$**	pos	308.0913±0.002
**GSH isotopologue+1**	pos	309.0943±0.002
**GSH isotopologue+2**	pos	310.0899±0.002
